# Polygenic Basis and Variable Genetic Architectures Contribute to the Complex Nature of Body Weight —A Genome-Wide Study in Four Chinese Indigenous Chicken Breeds

**DOI:** 10.3389/fgene.2018.00229

**Published:** 2018-07-02

**Authors:** Yangyang Yuan, Dezhi Peng, Xiaorong Gu, Yanzhang Gong, Zheya Sheng, Xiaoxiang Hu

**Affiliations:** ^1^Key Laboratory of Agricultural Animal Genetics, Breeding and Reproduction of Ministry of Education, College of Animal Science and Technology, Huazhong Agricultural University, Wuhan, China; ^2^State Key Laboratory for Agro-Biotechnology, China Agricultural University, Beijing, China; ^3^National Engineering Laboratory for Animal Breeding, China Agricultural University, Beijing, China

**Keywords:** genome-wide association study, haplotype-based association study, genetic architecture, body weight, polygenic basis, Chinese indigenous chicken

## Abstract

Body weight (BW) is one of the most important economic traits for animal production and breeding, and it has been studied extensively for its phenotype–genotype associations. While mapping studies have mostly aimed at finding as many loci as possible that contributed to the variation in BW, the role of other factors in its genetic architecture, including their frequencies in the population and their interactions, have been largely overlooked. To comprehensively characterized the genetic architecture of BW, we performed a genome-wide association study (GWAS) both at the single-marker and haplotype level on birds from four indigenous Chinese chicken breeds (Chahua, Silkie, Langshan, and Beard), rather than studying crosses between two founder lines. Additionally, samples from two more breeds (Red Junglefowl and Recessive White) were included to better reflect variable genetic characteristics across populations. Six loci were mapped in this study, revealing the polygenic basis underlying BW. Moreover, by further examining the frequencies of the significantly associated haplotypes in each subpopulation and their effect sizes, most of the loci were found to affect BW in the Beard chicken breed alone. Two loci in GGA9 and GGA27, however, had a common effect on BW across subpopulations, showing that different underlying genetic mechanisms contribute to the phenotypic variability. These findings, particularly the variable genetic architectures found in different loci, improve our understanding of the overall genetic contributions to the large variability in BW among Chinese indigenous chicken breeds. These findings thus will have important implications for future chicken breeding.

## Introduction

As a well-studied quantitative trait in chickens, body weight (BW) is not only a major breed characteristic but also a trait of economic importance. One of the most used study approaches is gene mapping. Phenotype to genotype associations have revealed substantial information regarding the polygenic basis of chicken BW. To date, several hundred quantitative trait loci (QTLs) affecting BW at different stages have been listed in chickenQTLdb (Hu et al., 2016). As sequencing has become a more routine study tool, comparative population genomics have further advanced our understanding from an evolutionary prospective (Rubin et al., 2010; Elferink et al., 2012; Qanbari et al., 2015). Although one earlier study found that the body composition of mice was determined by a very large number of loci, each with an infinitesimally small effect (Martinez et al., 2000), recent studies showed that some genes could still have more important roles than others in contributing to the phenotypic variability. Regardless, only a handful of genes have been found to be candidates with relatively large effects on BW in chickens (Rubin et al., 2010; Gu et al., 2011; Tang et al., 2011; Xie et al., 2012; Jia et al., 2016; Wang et al., 2016). One reason underlying this dilemma is likely the highly variable genetic architecture. Apart from the number of genetic variants affecting a trait, other factors, such as the frequencies in the population, the magnitude of the effect sizes and their interactions with each other, and the environment, are also important to a comprehensive understanding of the complex genetic architecture (Timpson et al., 2018).

The classical experimental design in mapping studies involves crosses between two founder lines, one of which is often a commercial line with limited genetic diversity. The abundant indigenous chicken breeds in China, which have a wide geographical distribution across the country and thus show remarkable differences in morphology, production and BW growth (Zhang et al., 2011), are valuable genetic resources to study polygenic basis and elucidate variable genetic architecture of the BW traits. Many QTLs have been repeatedly detected in several Chinese indigenous chicken breeds (Hu et al., 2016), which indicates a shared genetic basis. In contrast, due to the complex origin and demographic history of adaptation of the native birds (Miao et al., 2013), a few identified genes have common influences in several native breeds. One recent finding of a bone morphogenetic protein 10 (BMP10) mutation in Yuanbao chickens, which significantly decreases body length, reinforces this impression (Wang et al., 2016).

The aim of this study was to comprehensively characterize the genetic architecture affecting BW in Chinese chickens. We sampled birds from four Chinese indigenous breeds and recorded their BW from birth to 15 weeks of age. By performing a genome-wide association (GWA) study on both single-markers and haplotypes, we identified six loci contributing to the variation of BW in different breeds and characterized their frequencies across the subpopulations as well as the corresponding effect sizes. Epistatic scans further uncovered several pairs of interactive loci. These findings reveal that the complexity of BW not only originates from multiple loci contributing to the same trait but also the underlying variable genetic architectures. These will aid in the experimental design of future studies to provide a comprehensive understanding of the genetic contributions to phenotypic variance.

## Materials and Methods

### Animal Experimental Ethics

All animals used in the current study were cared for and used according to the guidances (HZAUMU2013-0005) approved by the Ethics Committee of Huazhong Agricultural University.

### Experimental Animals

The samples of our studies were collected from the National Chickens Genetic Resources (NCGR, Jiangsu Province)^1^, which is the off-site conservation base for 29 Chinese indigenous chicken breeds. In the NCGR, approximately 60 families are maintained for each generation of a single breed. Within each family, the mating ratio is 1–12. In this study, four indigenous breeds consisting of two typical low-body-weight breeds [Chahua chicken (C) and Silkie (S)] and two intermediate and high-body-weight breeds [Beard chicken (B) and Langshan chicken (L)], were included. Rather than directly sampling from the conserved population, birds were specifically bred for our study by performing artificial insemination on hens with sperm pools; in this way, birds used in this study were randomly selected from the original covserved population. After hatching these fertilized eggs to chicks, approximately 100 birds with approximately equal numbers of cocks and hens were phenotyped from each breed (**Supplementary Table S1**). Additionally, 4 Red Junglefowls (RJ) [data from (Elferink et al., 2012)] and 99 Recessive White chickens (RW) were used in the haplotype-based analyses.

### Phenotyping

Live BW was measured at hatch and every week until 15 weeks of age, after which all chickens were euthanized. For each BW trait of a single breed, boxplots were generated by R (3.3.0)^2^ (R Core Team, 2017) to screen for outliers. Records that were more than 1.5 times the interquartile range away from the lower or upper quartile of the boxplots were marked for further examinations. Such outliers were maintained only when they were consistently high across the growth phase; otherwise, the data points were eliminated from further analysis. The phenotypic records of eight individuals (2C, 4L, and 2B) were removed, as more than one-third of their data points failed the quality control.

### Genotyping

Blood samples were collected at 15 weeks of age. Genomic DNA was then extracted by the phenol-chloroform method and diluted to 50 ng/ml. Genotyping was performed using Illumina 60K Chicken SNP BeadChips (Groenen et al., 2011). Quality control was conducted on all 394 birds (after quality control of their phenotypic records) across four breeds by customized scripts in R (3.3.0) (R Core Team, 2017) using the following criteria: individual samples were excluded with call rates < 0.9; single-nucleotide polymorphisms (SNPs) were removed as a result of call rates < 0.9, minor allele frequency (MAF) < 0.05, or undetermined positions on the chromosome. For the Z chromosome, 1611 markers were excluded since they were falsely genotyped as heterozygous in female individuals.

After imposing the above constraints, 388 individuals (**Supplementary Table S1**) and 46211 SNP markers (**Supplementary Table S2**) were used for the next-step analyses.

### Genetic Diversity and Population Structure Analysis

PLINK (1.9) (Purcell et al., 2007) was used to evaluate the genetic diversity within each subpopulation. Firstly, we calculated the allele frequencies of both alleles at each locus using the command “--freq” and then obtained the proportion of polymorphic loci (P_poly_), whose MAF > 0.05, across all 46211 SNPs. Next, the pairwise linkage disequilibrium values (*r*^2^) from the command “--ld-window” were used to select the independent SNP sets of each subpopulation and to plot the pattern of linkage disequilibrium (LD) decay. Markers were determined to be “independent” when the *r*^2^ value between them was below 0.2. The expected heterozygosity values (*H*_e_) were derived at each of the independent SNP loci using the command “--hardy”; the Hardy–Weinberg equilibrium exact test *p*-values were acquired from the same command. Finally, we assessed the population structure by principal component analysis using TASSEL (5.2.30) (Bradbury et al., 2007).

### Statistical Analysis

Consequently, in addition to the two non-genetic factors “sex” and “birth-weight,” “breed” was included as the third fixed effect in the following models. To further correct for population stratifications, the polygenic effect as a random effect was also included. Therefore, the three fixed effects and the polygenic effect constituted the basic model for all of the following analyses done by the R package – GenABEL (Aulchenko et al., 2007b) throughout this study. (All scripts are available from the authors on request).

A two-step score test using mixed model and regression (GRAMMAR) (Aulchenko et al., 2007a) was adopted in GenABEL. Briefly, by implementing the kinship matrix from “ibs” into the function “polygenic” in GenABEL, we obtained the residuals from the model, which accounted for all the fixed effects, the polygenic effect, and covariates, if they were present. Then, these residuals were used as the dependent traits in a simple linear regression for single-marker, haplotype-based association, or epistatic scans.

#### Single-Marker Association Analysis

For the single-marker association study, the linear mixed model was constructed as follows:

(1) $$y=\mu +\text{F}\beta +X_{\text{S}}a_{\text{S}}+\text{G}+\varepsilon$$

Here, *y* is the phenotypic value, μ is the overall mean, *β* is the fixed effects, F is the design matrix of all three fixed effects, a_S_ is the marker genotype effect, and *X*_S_ is the vector of genotypes at the tested SNP, *G* is the random polygenic effect; its variance is defined as ∅$\sigma_{G}^{2}$, where ∅ is the kinship matrix from the whole-genome SNPs and $\sigma_{G}^{2}$ is the additive genetic variance due to the polygenes. In addition, the residual effects 𝜀 ~ (0, I$G_{2}^{\varepsilon }$).

In GenABEL, the first step is to build a kinship matrix ϕ with the whole-genome SNPs using the function “ibs,” then, by using “polygenic” and “mmscore,” we further estimated the genetic effects of each SNP.

Meanwhile, a second R package, FarmCPU (Liu et al., 2016), was also used here. In FarmCPU, rather than using the whole-genome SNPs, the kinship matrix ∅ was defined by a selected set of “pseudo quantitative trait nucleotides (QTNs).” Briefly, to remove the confounding between the tested SNP and both population structure and kinship, one fixed model and one random model were iteratively tested. The random model was used to select and evaluate the set of “pseudo QTNs” for every tested SNP, and the fixed model then fitted these “QTNs” to control false positives. Estimated genetic effects of the tested SNP were obtained, when a stage of convergence of the two models was reached after the iterative process.

The genomic inflation factor λ of both methods was further examined. Values of λ below 1.1 were considered acceptable, and the test statistics were further divided by λ to ensure that there were no indications of population stratification or cryptic relatedness in the final corrected dataset.

When multiple SNPs on the same chromosome were found to be significantly associated with the same trait, tests for their independence were performed. We first included the most significant SNP as an additional covariate in the model, and then we tested the remaining SNP(s) individually. If there were tested SNP(s) still showing significant association with the traits, the most significant SNP from this round of testing was also included as one covariate; the model then contained two SNPs as covariates. The testing continued until no significantly associated SNP was found [see model (2) below]. Last, we defined those remaining significant SNPs as independent signals, which led to our final result set.

(2) $$y=\mu +\text{F}\beta +\sum_{i=1}^{N}C_{i}\gamma_{i}+{\text{X}}_{s}a_{s}+\text{G}+\varepsilon$$

Here, *y*, μ, β, F, *a*_s_, *X*_s_, G, and 𝜀 are the same as described in model 1; *N* is the number of covariates included in the model, γ_i_ is the effect of the ith SNP as the covariate, and *C*_i_ is the vector of the corresponding covariate.

The genome-wide significance thresholds were defined using a randomization test based on 1,000 permuted datasets (Churchill and Doerge, 1994). Afterward, 1 and 5% significance thresholds of each trait were used to screen for significantly associated SNPs.

The proportion of the phenotypic variance explained by each of the significant SNPs was calculated as follows:

$$Var\%=\frac{2pqa^{2}}{Var_{y}}*100\%$$

Where *p* and *q* are the frequencies of the two alleles at the tested locus. As all of the final SNPs were associated with BW at multiple weeks of age, we used the phenotype that had the most significant association with the SNPs. Thus, *a* is the marker genotype effect estimated from the selected BW trait and *Var_y_* is the variance of the selected BW trait.

#### Haplotype-Based Association Analysis

To further explore the effects of haplotypes harboring the identified significant SNPs, the corresponding blocks were first defined. By employing Haploview 4.2 (Barrett et al., 2005), pair-wise LD *r*^2^ values were evaluated for each breed separately. Haplotype blocks were either defined directly by the default settings implemented in Haploview or defined as the clusters of SNPs that had *r*^2^ > 0.2 between its adjacent SNPs. Within these selected regions, SNPs were phased using fastPHASE 1.2 (Scheet and Stephens, 2006) and then the frequencies of all identified haplotypes were calculated. We retained those haplotypes that had frequencies ≥ 5% in at least one of the subpopulations for further association analyses.

The haplotype effects were evaluated at two levels based on model 3.

(3) $$y=\mu +\text{F}\beta +X_{h}a_{h}+\text{G}+\varepsilon$$

Here, *y*, μ, β, F, G, and 𝜀 are the same as described in model 1.

First, we tested the haplotypes separately to obtain the individual effects. For example, there were six haplotypes defined for BW_Q2 (**Supplementary Table S3**). When the effect of haplotype “CGA**G**” was to be estimated, two “alleles” were defined accordingly, one was this “CGA**G**” and all remaining haplotypes were defined as the other “allele.” Therefore, its effect could be evaluated as the allele substitution effect. In addition, *a*_h_ is the estimate of the tested haplotype, while *X*_h_ is a column vector filled with the counts of the selected haplotype (0, 1, or 2). Bonferroni correction was used to obtain the threshold and the total test number was the sum of the tested haplotypes across the six loci.

Second, the effects of those significantly associated haplotypes were further estimated in their diploid form. Using haplotype “CGA**G**” in BW_Q2 as an example, the estimates of homozygotes “CGA**G**/CGA**G**” were evaluated in this step. Here, the coefficient vector *X*_h_ consists of values inferring the corresponding genotypes of each sample (e.g., coefficient “66” means the corresponding individual had haplotype “CGA**G**” on both chromosomes, and there were tens of different genotypes for one locus); *a*_h_ is the estimates of the two combined haplotypes. Again, Bonferroni correction was used. The total test number was the number of tested loci, which equaled six.

#### Epistatic Analysis

To perform the variance-heterogeneity genome-wide association study (vGWAS), we transformed the phenotypes following an inverse-normal transformation (customized R scripts can be found in **Supplementary Data Sheet S1**). Then, the values were squared before we used them in the standard single-maker GWA analyses by GenABEL, as model 1 [details can be found in Yang et al. (2012), Shen et al. (2014)].

Significant results from the vGWAS together with selected SNPs from the GWA analyses (a total of 10 SNPs) were further examined individually against the whole-genome SNP set, based on a two-locus model, to scan for the potential G × G pairs.

(4) $$y=\mu +\text{F}\beta +\text{G}+X_{1}a_{1}+X_{2}a_{2}+\varepsilon$$

(5) $$y=\mu +\text{F}\beta +\text{G}+X_{1}a_{1}+X_{2}a_{2}+\text{E}a_{1\times 2}+\varepsilon$$

Here, *y*, μ, β, F, G, and 𝜀 are the same as model 1. The null model (model 4) included the marker genotype effects of two loci, where *X*_1_ and *X*_2_ are the coefficient vectors of additive effects, and *a*_1_ and *a*_2_ are the effect estimates of the two tested SNPs, respectively. The full model (model 5) fitted an extra interactive term with *E*, the design matrix for the interactive effect between the two loci, and *a*_1 × 2_, the estimated epistatic genetic effect. By comparing the residuals from these two models, *F* statistics were calculated to evaluate the significance of the effects. Accordingly, permutation tests based on 1000 randomized datasets were performed to empirically derive the significance for each of the 10 targeted SNPs.

### Candidate Gene Search and Gene Ontology (GO) Analysis

Based on the pairwise *r*^2^ values calculated in the earlier steps, the average lengths between two markers, when their *r*^2^ dropped to 0.2, were obtained (**Table 1**). Given that *r*^2^ descreased over the longest distance (72 Kb) in Silkie chicken, the candidate regions were thus determined to 72 Kb upstream and downstream of the significant SNPs. Therefore, candidate genes were searched within these defined regions using ENSEMBL biomart^3^ (Kinsella et al., 2011). GO enrichment analysis on the identified candidate genes was further carried out using DAVID^4^.

**Table 1:** Estimates of genetic diversity within each subpopulation.

Breeds	*N*_samples_	Estimated on all markers			Estimated on independent markers		
		*N*_all_M_^1^	*P*_poly_^2^	*P*_H-W_^3^	*N*_indep_M_^4^	*L*_r2 = 0.2_^5^	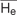 ^6^
Chahua	97	46211	84.66%	99.27%	5980	7.5	0.299
Silkie	100		80.31%	99.45%	4726	72	0.333
Langshan	96		84.08%	99.36%	4926	62.5	0.309
Beard	95		94.82%	99.27%	13225	3.5	0.362

*^*1*^N_*all_M*_ indicates means the number of all markers after quality control.*

*^*2*^P_*poly*_ indicates the percentage of polymorphic loci, with minor allele frequencies more than 0.05.*

*^*3*^P_*H-W*_ indicates the percentage of SNP loci conformed to Hardy–Weinberg expectations.*

*^*4*^N_*indep_M*_ indicates the number of independent markers in each subpopulation.*

*^*5*^L_*r2* = *0.2*_ indicates the average physical distance (Kb) between two adjacent markers when their r^*2*^ drop to 0.2.*

*^*6*^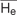 indicates the average expected heterozygosity.*

## Results

### Genetic Diversity of Beard Chickens Is Different From That of Other Subpopulations

**FIGURE 1:**
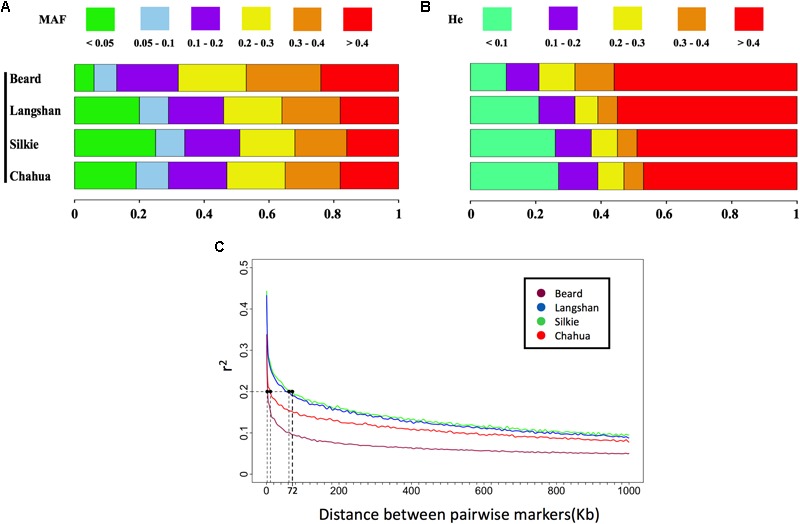
Genetic diversity of four subpopulations estimated by MAF, *H*_e_, and LD decay. **(A)** Distributions of minor allele frequency (MAF) estimated from the whole-genome single-nucleotide polymorphism (SNP) set in each subpopulation. Rectangles of different colors indicate different clusters of MAF, while rectangles of different lengths indicate the proportions of markers within different clusters. **(B)** Distributions of expected heterozygosity (*H*_e_) estimated from the derived independent SNP set in each subpopulation. Rectangles of different colors indicate different clusters of *H*_e_, while rectangles of different lengths indicate the proportins of markers within different clusters. **(C)** Decay of linkage disequilibrium (LD) of the four subpopulations across the genome. The pairwise linkage disequilibrium (*r*^2^) is plotted against the corresponding physical distances. When *r*^2^ drops to 0.2, the longest interval between two SNPs is the 72 Kb observed in Silkie (shown with dotted lines on graph).

As a result of random sampling from the original conserved populations, the observed genotypes at approximately 99% of SNP loci conformed to Hardy–Weinberg expectations in each subpopulation (**Table 1**). The distributions of MAF and *H*_e_ estimated from the whole-genome SNP set in the four subpopulations are presented in **Figures 1A,B**. While Chahua, Silkie, and Langshan breeds show similar distributions of these two parameters, Beard displays a rather different pattern. We observed a decreased proportion of MAF and *H*_e_ between 0 to 0.1 in Beard chickens, which revealed that a relatively smaller part of Beard’s genome has low levels of heterozygosity. The LD-decay pattern (**Table 1** and **Figure 1C**) also shows that *r*^2^ decreases over shorter distances (an average of 3.5 Kb) in Beard than in the other three subpopulations. Overall, there was a clear difference in the level of genetic diversity between Beard and the other three subpopulations.

### Large BW Variations Were Observed Among These Indigenous Breeds

**FIGURE 2:**
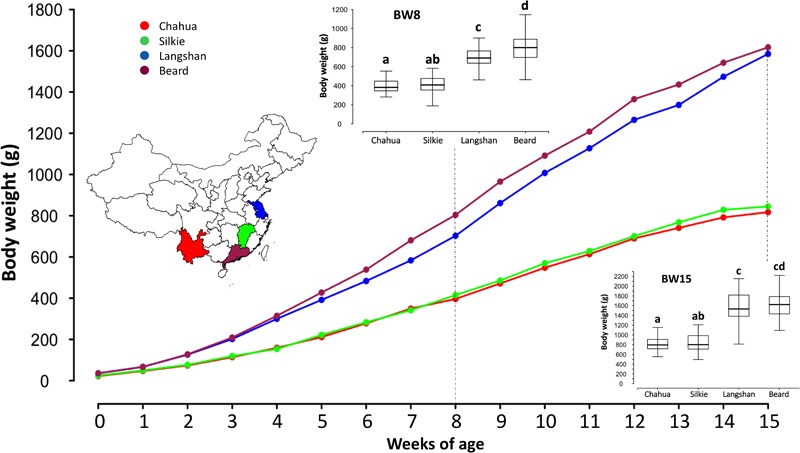
Growth curves of the four studied subpopulations from birth to 15 weeks of age. *X*-axis of the main plot shows chickens at different weeks of age, while *y*-axis presents their mean body weights. The inset map of China at the left side is marked with different colors to show the geographical origins of the four subpopulations. Boxplot insets, as two examples, show the mean differences of four subpopulations at 8 and 15 weeks of age. Letters a–d in the boxplots indicate the statistical differences between subpopulations. Subpopulations with different letters have significantly different means (*p* < 0.05). For example, at 8 weeks of age, the mean body weights between Chahua and Silkie are not significantly different, as they both are labeled with the letter “a.” On the contrary, the mean body weight of Langshan is significantly different from that of Beard, as they are labeled with different letters.

After quality control, 388 birds from four Chinese native chicken breeds were included in this study and their live BW measurements were recorded from birth to 15 weeks of age. The descriptive statistics of the phenotypic measurements of each subpopulation are given in **Supplementary Table S4** and their growth curves are presented in **Figure 2**. In agreement with their adult weight [mean values of BW at 300 days of age recorded in *Animal Genetic Resources in China-Poultry* (Zhang et al., 2011)], the 15-week BWs of Beard and Langshan chickens were significantly heavier than those of Chahua and Silkie (*p* < 0.01, *t*-test). While no significant differences were found between Chahua and Silkie throughout all examined weeks, there were differences between Beard and Langshan chickens from weeks 3 to 14. Therefore, these large variations in BW provided the basis for our next-step analysis.

### Six Loci Were Found to Be Significantly Associated With BW Across Different Growth Stages in This Population

**FIGURE 3:**
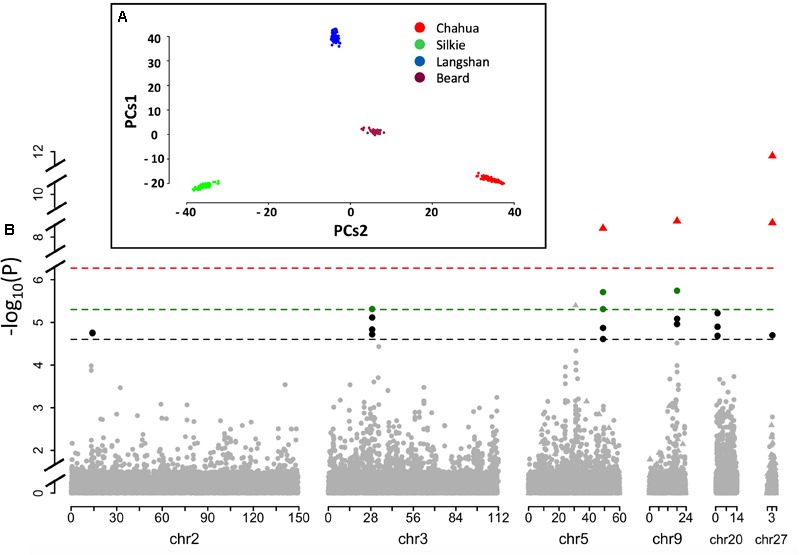
Quantitative trait loci (QTL) profile of the six identified loci across the genome. **(A)** Mapping samples to the space of the first two Principle Components (PCs1 vs. PCs2) resulting from analysis of genomic kinship. Dots in different colors represent samples from different subpopulations. **(B)** Schematic of the results from the single-marker association study based on GenABEL and FarmCPU methods. The dashed lines in black, and green denote the 5 and 1% genome-wide significance, respectively, based on package GenABEL. The red line shows the 1% genome-wide significance based on package FarmCPU. Dots represent the results from GenABEL, while triangles represent the results from FarmCPU.

The principal component analysis of our dataset using the first two components showed that these four subpopulations had distinct genetic backgrounds (**Figure 3A**). Based on thresholds determined by permutation tests, significantly associated SNPs found by R package *GenABEL* were clustered into 13 QTLs (**Supplementary Table S5**). Of these 13 QTLs, five were found to be associated with more than one BW trait and thus were retained for further testing. Moreover, a second R package, *FarmCPU* was employed to perform a parallel GWA analysis. Three significant signals co-localized with QTLs detected by *GenABEL*, which were also included in the final result set (**Supplementary Table S5**). After testing for their independence, a total of 8 SNPs clustered into six independent QTLs on six chromosomes were identified at two significance levels in our single-marker association analysis (**Table 2** and **Figure 3B**). These loci were named as a combination of BW_Q (QTLs associated with BW) and the chromosome number where the QTL was located. Moreover, when multiple independent QTLs co-localized on the same chromosome, ordered letters in the alphabet were added at the end to indicate their sequential position along the same chromosome.

**Table 2:** Significant QTLs affecting body weight traits in this study.

Name	SNP	Chromosome	Position (bp)^1^	Trait^2^	Var%^3^
BW_Q2	rs15896134	2	14068213	BW4**^G^**	1.0
				BW6**^G^**	1.3
BW_Q3	rs14332618	3	28927355	BW9^**G**^	1.5
				BW10**^∗G^**	1.8
				BW11**^G^**	1.6
				BW12**^G^**	1.7
BW_Q5	rs14545931	5	49062749	BW8**^G^**	1.1
				BW10**^∗G^**	1.2
				BW11**^G^**	1.1
				BW12**^∗G^**	1.3
				BW12**^∗F^**	1.0
BW_Q9	rs313615987	9	18601322	BW15**^∗G^**	1.3
				BW15**^∗F^**	1.0
	rs14678295	9	18643628	BW14**^G^**	1.4
BW_Q20	rs315743526	20	1301395	BW12**^G^**	1.0
				BW13**^G^**	1.2
				BW15**^G^**	1.0
BW_Q27	rs16719177	27	3326082	BW14**^∗F^**	1.5
				BW15**^∗F^**	1.0
	rs16040772	27	3335587	BW7**^G^**	1.0

*^*1*^Position from the assembly of Gallus gallus 5.0.*

*^*2*^“∗” denotes 1% genome-wide significance, while the remaining loci were the 5% genome-wide significance.; G denotes results obtained from GenABEL, F denotes results from FarmCPU.*

*^*3*^The percentage of phenotypic variation explained by the detected SNP.*

All six QTLs were found to be associated with BW at several weeks of age. Especially, two QTLs, BW_Q9 and BW_Q27, were comprised of two correlated SNPs that were only tens of Kbs apart. In addition, more QTLs, with larger average effects, were found for growth at intermediate and late growth phases than for growth at early phases. Each QTL explained only a small proportion of the phenotypic variances, which further revealed the highly polygenic nature of the BW trait.

### Haplotypes Within the Six QTLs Revealed Both Unique and Shared Genetic Basis Underlying the Large Variations in BW Among the Four Subpopulations

**FIGURE 4:**
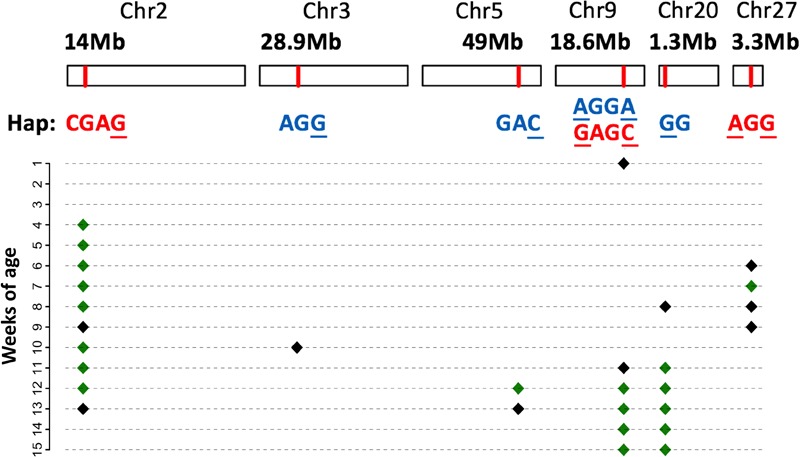
Significant haplotype profile across the genome. The physical position of each QTL is shown at the top; below is the corresponding significant haplotype within each locus. Core SNPs are marked with the underscores. Red letters mean that the corresponding haplotypes increase body weight, while blue letters denote an effect of decreasing the body weight. Their manifested body weight traits at different weeks of age are plotted below, where black and green diamonds denote the 5 and 1% genome-wide significance, respectively.

Next, we examined the identified loci based on haplotypes around the significant SNPs (i.e., core SNPs) listed in **Table 2**. The effects of all six QTLs were confirmed to be significant in the haplotype-based association (**Figure 4**). To further clarify the patterns of haplotype frequencies across different subpopulations, additional data from 4 RJ and 99 RW chickens were used.

In general, the identified haplotypes were associated with BW traits for a longer period of time than those found in the single-marker association analysis. In concordance with this result, the estimated effects of these haplotypes were larger than those of the corresponding significant SNPs. For each locus, multiple haplotypes were defined across 2–6 SNPs. Nevertheless, except for BW_Q9, there was only one haplotype that was found to be significantly associated with the BW traits in the other five QTLs. In addition, the phenotypic values of individuals, which were homozygous for the identified haplotype, significantly deviated from the mean values.

Compared with other haplotypes within BW_Q2, the identified haplotype in this locus (hereafter called BW_H2, similar to the rules used in naming QTLs) had a positive additive effect on BW throughout 10 weeks of growth (**Table 3**). However, rather than being the major haplotype in the two high BW subpopulations, BW_H2 was uniquely found in the Beard chickens. Additionally, while the core SNP was polymorphic in both RJ and RW samples, no birds in RJ and only a few in RW had this haplotype (**Supplementary Table S3**). Therefore, we infer that this haplotype is newly evolved in Beard chickens, which shows its unique potential in the genetic improvement of BW.

**Table 3:** Estimated effects of the significant haplotypes and their corresponding homozygotes within six identified QTLs.

Name	Haplotype^1^	Trait	EffectH ± SD^2^	*P*-value^3^	EffectD ± SD^4^	*P*-value^3^
BW_H2	CGA**G**	BW4	16.3 ± 3.9	3.9E-5^∗^	41.8 ± 11.3	2.4E-4^∗^
		BW5	20.4 ± 5.4	1.9E-4^∗^	51.2 ± 15.7	1.2E-3^∗^
		BW6	31.1 ± 7.2	2.2E-5^∗^	91.8 ± 20.7	1.2E-5^∗^
		BW7	36.3 ± 9.6	1.9E-4^∗^	104.4 ± 27.1	1.4E-4^∗^
		BW8	47.1 ± 11.2	3.3E-5^∗^	137.8 ± 32.1	2.3E-5^∗^
		BW9	46.2 ± 13.3	5.8E-4	133.4 ± 38.6	6.1E-4^∗^
		BW10	62.2 ± 15.4	6.6E-5^∗^	182 ± 44.6	5.5E-5^∗^
		BW11	61.1 ± 16.9	3.4E-4	176.1 ± 48.7	3.4E-4^∗^
		BW12	71.4 ± 19.4	2.7E-4^∗^	170.2 ± 56.5	2.8E-3
		BW13	72.6 ± 20.7	4.9E-4	172.2 ± 60.4	4.6E-3
BW_H3	AG**G**	BW3	/	/	-15 ± 5.1	3.3E-3
		BW5	/	/	-28.6 ± 10.6	7.4E-3
		BW8	/	/	-72.8 ± 22.2	1.1E-3^∗^
		BW9	/	/	-92.8 ± 26.2	4.5E-4^∗^
		BW10	-38.7 ± 12.1	1.5E-3	-107.8 ± 30.7	5E-4^∗^
		BW11	/	/	-112.1 ± 33.5	9E-4^∗^
		BW12	/	/	-127.7 ± 38.3	9.3E-4^∗^
		BW13	/	/	-136.6 ± 40.7	8.7E-4^∗^
		BW14	/	/	-137.5 ± 43.9	1.8E-3
		BW15	/	/	-152.3 ± 46.8	1.2E-3^∗^
BW_H5	GA**C**	BW6	/	/	-58 ± 19.6	3.3E-3
		BW7	/	/	-69.2 ± 26	8.2E-3
		BW8	/	/	-91.1 ± 30.1	2.6E-3
		BW9	/	/	-105 ± 35.5	3.3E-3
		BW10	/	/	-133.8 ± 41.5	1.3E-3^∗^
		BW11	/	/	-158 ± 45.2	5.2E-4^∗^
		BW12	-70.1 ± 18.8	2.1E-4^∗^	-186.2 ± 51.9	3.7E-4^∗^
		BW13	-65.3 ± 20.1	1.2E-3	-189.4 ± 55.3	6.7E-4^∗^
		BW14	/	/	-213.2 ± 59	3.48E-4^∗^
		BW15	/	/	-240.1 ± 62.8	1.5E-4^∗^
BW_H9a	**A**GG**A**	BW11	-35.7 ± 10.2	5.5E-4	82.2 ± 27.4^**ab**^	2.9E-3
		BW12	-43.7 ± 11.8	2.3E-4^∗^	111.1 ± 31.3^**ab**^	4.3E-4^∗^
		BW13	-46.6 ± 12.5	2.3E-5^∗^	104.6 ± 33.2^**ab**^	1.7E-3
		BW14	-54.5 ± 13.3	5.3E-5^∗^	141.5 ± 35.1^**ab**^	6.8E-5^∗^
		BW15	-55.8 ± 14.3	1.1E-4^∗^	149.4 ± 37.7^B^	9E-5^∗^
BW_H9b	**G**AG**C**	BW13	/	/	310.5 ± 109.8	5E-3
		BW14	90.3 ± 27.5	1.1E-3	385.9 ± 115.9	1E-3^∗^
		BW15	/	/	386.1 ± 124.6	2.1E-3
BW_H20	**G**G	BW8	-27.6 ± 8.6	1.4E-3	/	/
		BW11	-68.4 ± 15.5	1.4E-5^∗^	-94.2 ± 31.7	3.2E-3
		BW12	-74.1 ± 17.8	3.9E-5^∗^	-130 ± 36.5	4.2E-4^∗^
		BW13	-59.1 ± 14.7	6.8E-5^∗^	-138.7 ± 38.6	3.8E-4^∗^
		BW14	-64.7 ± 16.7	1.3E-4^∗^	-135.6 ± 41.6	1.2E-3^∗^
		BW15	-49 ± 12.8	1.5E-4^∗^	-146.5 ± 44.3	1E-3^∗^
BW_H27	**A**G**G**	BW4	/	/	21.8 ± 8	7E-3
		BW5	/	/	31.9 ± 11.1	4.2E-3
		BW6	17.8 ± 5.4	1.1.E-3	45 ± 14.7	2.4E-3
		BW7	28.6 ± 7.2	8.2E-5^∗^	72.4 ± 19.2	1.9E-4^∗^
		BW8	29.1 ± 8.4	5.9E-4	69.6 ± 22.5	2.2E-3
		BW9	34.1 ± 9.9	6.8E-4	/	/

*^*1*^Shows significant haplotype detected within each QTL, core SNPs are marked in bold and with the underscores.*

*^*2*^Presents estimates of the effects of haplotypes.*

*^*3*^“∗” denotes 1% test-wide significance, while the remaining loci were the 5% test-wide significance.*

*^*4*^Presents estimates of the effects of the homozygous haplotypes. Specifically, letter ab means that the denoted estimates are of the combined effects of BW_H9a and BW_H9b.*

It is interesting that half of the QTLs had haplotypes (BW_H3, BW_H5 and BW_H20) that were found to significantly decrease the BW (**Table 3**). More notably, apart from BW_H20 that also represented a small proportion in Langshan (5%), the haplotypes were again represented by one major haplotype (>20%), which was uniquely found in Beard chickens. In four RJ samples, the core SNPs were all fixed for the allele other than those in the identified haplotypes. Consequently, none of these haplotypes were found in RJ. However, unlike BW_Q2, they are quite common in RW (**Supplementary Table S3**). This might indicate that, regardless of its decreased effects on BW in Beard and RW chickens, it is still preferable in selection due to its association with other traits.

**FIGURE 5:**
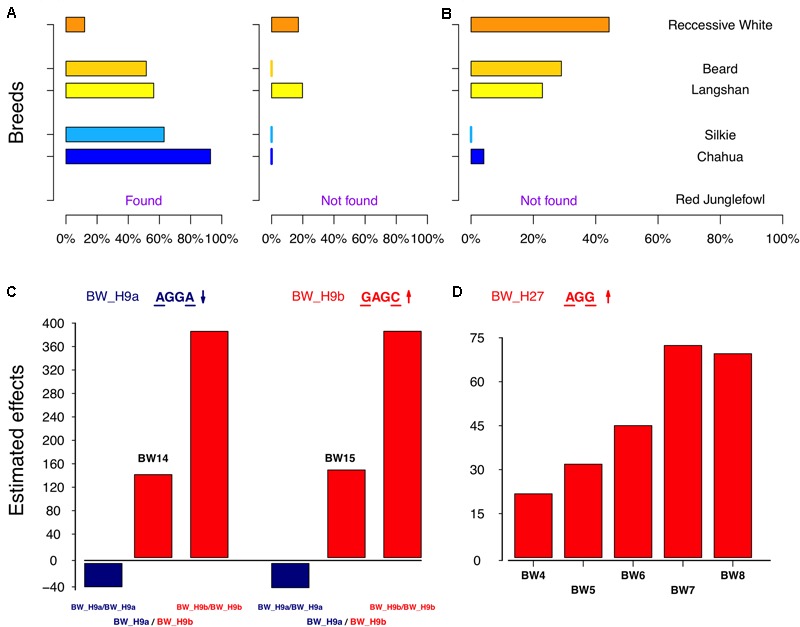
Genetic characteristics of the significant haplotypes in BW_Q9 and Q27. **(A,B)** Distribution of the significant haplotypes from BW_Q9 and Q27 in six subpopulations. **(C)** Estimated effects of the combined two haplotypes, BW_H9a and BW_H9b, on body weight at 14 and 15 weeks of age. **(D)** Estimated effects of the homozygous BW_H27 on body weight from 4 to 8 weeks of age.

The remaining loci were those containing two core SNPs. The identified haplotype, BW_H27, had a significantly positive effect on BW, compared with that of other haplotypes in locus BW_Q27 (**Figure 5D**). As expected, BW_H27 shared much higher frequencies in Langshan (23%), Beard (29%), and RW (44%) than it did in Chahua (4%), Silkie (0%), and RJ (not found) (**Figure 5B**). This observation suggested that this locus is a common contributor to BW in these four indigenous breeds (Chahua, Silkie, Langshan, and Beard). Unlike any loci described above, we found two haplotypes that were significantly associated with BW traits in BW_Q9 (**Figure 5A**). Moreover, the effects of these two haplotypes, BW_H9a and BW_H9b, were of different directions. BW_H9a had a negative additive effect, and was found to be associated with BW traits for five consecutive weeks during the late growth phase. However, at 14 weeks of age, when both BW_H9a and BW_H9b were significantly associated with the BW, the effect size of BW_H9b was larger than that of BW_H9a (**Figure 5C**). Additionally, haplotype BW_H9b was only found in Langshan and RW. Therefore, locus BW_Q9 contributes to decrease BW in Chuahua, Silkie, and Beard, while it is more likely to increase BW in Langshan. Therefore, although BW_Q27 and BW_Q9 both had effects on all four indigenous breeds, their underlying genetic architectures are not the same.

### Detected Significant Interactive Pairs Infer a Role of Epistasis in the Genetic Basis of BW

First, we screened for vGWAS signals, which suggest loci that are under the genetic control of plasticity through G × G or G × E interactions. Six loci were detected, two of which overlapped with the core SNPs found in BW_Q5 and BW_Q9 (**Table 4**). Then, together with six core SNPs found in the single-marker association analysis, a total of 10 SNPs (4 from vGWAS results) were utilized in the two-locus epistatic model. An exhaustive two-locus interaction scan was performed between the targeted SNPs (individually) and the remainder of the full SNP dataset. Based on permutation tests, interactive pairs, including three loci that originated from the vGWAS scan, were detected at a 10% genome-wide suggestive significance level (**Supplementary Table S6**). Moreover, 6 SNPs from 5 loci were found to significantly interact with the core SNP, rs15896134 in BW_Q2 (5% genome-wide significance). The combinations of genotypes between the detected two loci as well as their corresponding phenotypes are presented as boxplots in **Supplementary Figure S1**. This finding might imply a role of epistasis in driving haplotypes uniquely evolved in one of our subpopulations, such as BW_H2 in Beard chickens. However, we do advise caution in interpreting these results, as the significance of epistatic interactions were mostly due to deviated phenotypic values of a few individuals.

**Table 4:** Single-nucleotide polymorphisms (SNPs) of significant variance-heterogeneity detected by vGWAS in this study.

SNP	Chromosome	Position (bp)^1^	Trait	*P*-value^2^
rs16225434	3	7928979	BW2	1.5E-5
rs14545931 (BW_Q5)	5	49062749	BW10	1.8E-5
			BW12	1.8E-6^∗^
rs313615987 (BW_Q9)	9	18601322	BW15	8.9E-7^∗^
rs315546657	10	13131038	BW4	7.2E-6
rs14024825	11	10905011	BW10	1.1E-5
rs14274361	20	6829862	BW1	2.4E-6^∗^

*^*1*^Position from the assembly of Gallus gallus 5.0.*

*^*2*^“∗” denotes 1% genome-wide significance, while the remaining loci were the 5% genome-wide significance.*

## Discussion

To explore the genetic basis underlying the large variation in BW among Chinese indigenous chicken breeds, we herein described a GWA study in a population that consisted of four Chinese indigenous subpopulations. Due to the small sample size and the mixed population structure, a stringent standard was applied to control the stratification. Significant results either were determined by two analytic methods or were found to be associated with BW traits at different weeks of age. Based on the idea that the haplotype would be in greater LD with the causal mutation than a single SNP would (Cedric et al., 2013), haplotype-based association analyses were further performed. Then, not only were all loci validated, but the power of the analyses was increased so that larger effects and more associated traits were obtained (**Table 3**). Finally, a total of six QTLs with seven underlying haplotypes were found to be significantly associated with BW traits from birth to 15 weeks of age.

We performed candidate gene searches and preliminary GO analysis within both the single and epistatic QTL regions. The QTL regions surrounding the significant SNPs were defined to a relatively small region (2 × 72 Kb) based on the average LD decay distance between two markers in Silkie, which was the longest among these four subpopulations. Therefore, a small number of candidate genes were identified and no enriched pathway was found. Because functional validation of these candidate genes was beyond the scope of the current study, we did not list all of them in the “Results” section, but we have included some of the promising candidates in the following discussion. These candidates had been functionally studied in previous reports.

Given the large confidence intervals normally obtained in classical QTL scans, it is not surprising that all six loci co-localize with the QTLs from previous studies in chickenQTLdb (Hu et al., 2016). The earlier studies in Chinese indigenous chickens had also detected BW_Q9 in Xinghua chicken (Fang et al., 2010) and BW_Q27 in Beard chicken (Sheng et al., 2013), which further confirmed their common role in affecting BW among different breeds. Meanwhile, candidate gene searching within the regions that were 72 Kb (defined in **Figure 1C**) upstream and downstream of the detected SNPs also identified two important functionally related genes. One is delta like non-canonical Notch ligand 1 (*DLK1*), which is 32 Kb upstream of the identified core SNP in BW_Q5. Previous studies had indicated its association with muscle tissue development in chickens (Shin et al., 2008) and obesity in humans (Persson-Augner et al., 2014). The other is growth differentiation factor 5 (*GDF5*), which contained the core SNP in BW_Q20 as an intron variant. Its effect on skeletal development in chickens (Francis-West et al., 1999) and adipogenesis in mice (Hinoi et al., 2014) suggests an important role in affecting BW.

The important contribution of epistasis to quantitative traits had long been established in several organisms (Carlborg et al., 2006; Wei et al., 2014; Mackay, 2015; Forsberg et al., 2017). Recently, scaning for genetic variance-heterogeneity (vGWAS) had been proposed as an effective method to reveal epistatic effects (Shen et al., 2014; Forsberg et al., 2015). In the present study, we also performed the epistatic scans first by vGWAS and then by the classical two-locus model. In the exhaustive two-locus epistatic scans, while half of the six genome-wide significant vGWAS results were paired with their suggestive significant interactive loci, only BW_Q2 from the GWA study results was identified with its significant interactive pairs. Although a number of different mechanisms other than epistasis can also lead to such genetic variance-heterogeneity (Struchalin et al., 2010; Rönnegård and Valdar, 2011, 2012), in our study, we did observe a substantial proportion of the vGWAS results showing significant gene–gene interaction effects on the phenotypes. Moreover, known candidate genes, such as ATP binding cassette subfamily D member 2 (*ABCD2*) (Liu et al., 2012), nuclear receptor interacting protein 1 (*NRIP1*) (Park et al., 2011), KIT proto-oncogene receptor tyrosine kinase (*KIT*) (Gutierrez et al., 2015) and butyrylcholinesterase *(BCHE)* (Lima et al., 2013), had been located in the vicinity of several significant interactive loci. However, we do advise caution in interpreting these results, as our study design is not ideal for such tests.

Studies aimed at revealing the major variant for BW trait had found that multiple important genes or pathways were responsible for the high variability in BW in Chinese indigenous chicken breeds. For example, BMP10 accounted for more than 20% of the phenotypic variation in the Yuanbao chicken (Wang et al., 2016). miR-16 in one major QTL from Xinghua chicken was found to decrease BW by inhibiting cell proliferation (Jia et al., 2016). Ubiquitin mediated proteolysis, identified from several pairs of interactive loci, played an important role in regulating BW in Beard chickens (Sheng et al., 2013). Nevertheless, most of these findings originated from a single indigenous breed, and there is a low degree of overlap between the results. This led to an assumption that the major contributor to BW in different breeds might not be the same. GO analysis in our study seems to support this assumption, since no significant enrichments of the candidate genes were revealed (detailed results not shown). Additionally, by further examining the significant haplotypes within each subpopulation, more loci had effects on BW in a single breed than had a common role in regulating BW across all breeds.

In concordance with the relatively higher level of genetic diversity observed in Beard chickens, four out of the six identified QTLs were the breed-specific loci, in which the significant haplotypes were uniquely found in Beard chickens (**Supplementary Table S3**). More notably, BW_Q3, 5, and 20 were transgressive loci, i.e., the haplotypes having negative effects on BW were of significantly higher proportion in the high BW breed (Beard) of this study. Similar findings had been observed in other chicken QTL scans (Abasht et al., 2006), even in one population under intense artificial selection for BW (Sheng et al., 2015), which suggested the presence of transgressive loci. One possibility underlying such observations would be epistasis, where the effects of these transgressive loci depended on the genetic context in which they resided. In our vGWAS analysis, BW_Q5 showed variance heterogeneity, suggesting that this locus was a candidate of G^∗^E or G^∗^G interactions. However, the sample size and marker density in our study limited our ability to find its interactive pair in the exhaustive two-locus scan. Another potential explanation would be that these loci are pleiotropic for traits that are negatively correlated with BW, similar patterns have been found in some plant studies (Qian et al., 2016; Li et al., 2018). Therefore, regardless that their effects on BW seemed to be against the selection in this population, the overall fitness could still increase as they might have positive effects (in terms of selection) on other trait(s). Further work, (e.g., more phenotyping) is needed to dissect the underlying mechanisms.

Unlike the four QTLs discussed above, BW_Q9 and Q27 were the breed-shared loci that were associated with BW across the four subpopulations. Earlier studies in Chinese indigenous chickens had already detected BW_Q9 in the Xinghua breed (Fang et al., 2010) and BW_Q27 in Beard chicken (Sheng et al., 2013), which further indicates the common role of these two loci in different breeds. However, by further examining the frequencies of their significant haplotypes across the four subpopulations as well as in the two comparisons, RJ and RW samples, the underlying genetic patterns of these two loci were not the same. Haplotype BW_H27, which increased BW, was present in greater proportions in the high BW subpopulations than in the low BW subpopulations. Locus BW_Q9 had two significant haplotypes that were of opposite effects, so that different combinations of these two haplotypes would contribute differently to the BW. Most studies that dissect the genetic architecture of a complex trait, such as BW, focus on the number of genetic variants affecting the trait and their physical positions in the genome. Herein, we showed an example where the additional information on the frequencies of underlying genetic variants across different subpopulations, their magnitude of effects, and gene–gene interactions provided an in-depth understanding of the genetic architecture of a complex trait, which will be important to future advances in animal breeding.

In summary, we performed a GWA study across four Chinese indigenous chicken breeds. The single-marker association study identified six QTLs that were significantly associated with BW from birth until 15 weeks of age, two of which (BW_Q9 and Q27) co-localized with known QTLs found in Chinese indigenous breeds. Additionally, four out of the six QTLs were polymorphisms in Beard chickens alone, thus, they were breed-specific loci that contributed to BW variability in one breed. This is probably caused by the complex origin and demographic history of domestic chickens (Miao et al., 2013). While most mapping studies have focused on the number of genetic variants that were responsible for the phenotypic variability, we have further examined their frequencies and the magnitude of effects at the haplotype level. The breed-shared loci BW_Q9 and Q27 showed distinct underlying genetic patterns, which suggests not only a polygenic basis but also a variant genetic architecture leading to the complex quantitative characteristics of BW.

## Data Availability Statement

The genotype and phenotype datasets of this study are provided as the **Supplementary Data Sheets S2, S3**.

## Author Contributions

ZS, XH, and YG conceived and designed the experiments. DP, XG, and ZS collected the samples and performed the experiments. YY and ZS analyzed the data and drafted the manuscript. XH and YG revised the manuscript. All authors read and approved the final manuscript.

## Conflict of Interest Statement

The authors declare that the research was conducted in the absence of any commercial or financial relationships that could be construed as a potential conflict of interest.

## Footnotes

1 http://www.genebank.org.cn/read.asp?id=1

2 https://www.r-project.org/

3 http://www.ensembl.org/biomart

4 https://david.ncifcrf.gov/

## Supplementary Material

FIGURE S1 Two-locus genotype-phenotype map for all significant interactive pairs. *X*-axis is the genotype combination of the two tested loci, while *y*-axis presents the residuals of phenotypes after correction for fixed and random effects. Numbers shown at the top in blue are the individual counts for each genotype combination.

TABLE S1 Data structure of the four subpopulations.

TABLE S2 Summary statistics of SNP markers acorss whole-genome in this dataset.

TABLE S3 Distribution of main haplotypes within each QTL across six sub-populations.

TABLE S4 Descriptive statistics of the body weight traits in four sub-populaitons.

TABLE S5 A full list of detected SNPs from single-marker association analyses.

TABLE S6 Significant SNP pairs from the exhaustive two-loci epistatic san in this study.

DATA SHEET S1 The R code for transforming the raw phenotypes for the vGWAS analysis.

DATA SHEET S2 The genotype datasets of this study.

DATA SHEET S3 The phenotype datasets of this study.
