# Supplementary material for: Polygenic Basis and Variable Genetic Architectures Contribute to the Complex Nature of Body Weight —A Genome-Wide Study in Four Chinese Indigenous Chicken Breeds
Source: Front Genet. 2018 Jul 2;9:229. doi: 10.3389/fgene.2018.00229 (PMC6036123; doi:10.3389/fgene.2018.00229)
Supplement: FIGURE S1 — Two-locus genotype-phenotype map for all significant interactive pairs. X-axis is the genotype combination of the two tested loci, while y-axis presents the residuals of phenotypes after correction for fixed and random effects. Numbers shown at the top in blue are the individual counts for each genotype combination. [file Image_1.PDF]

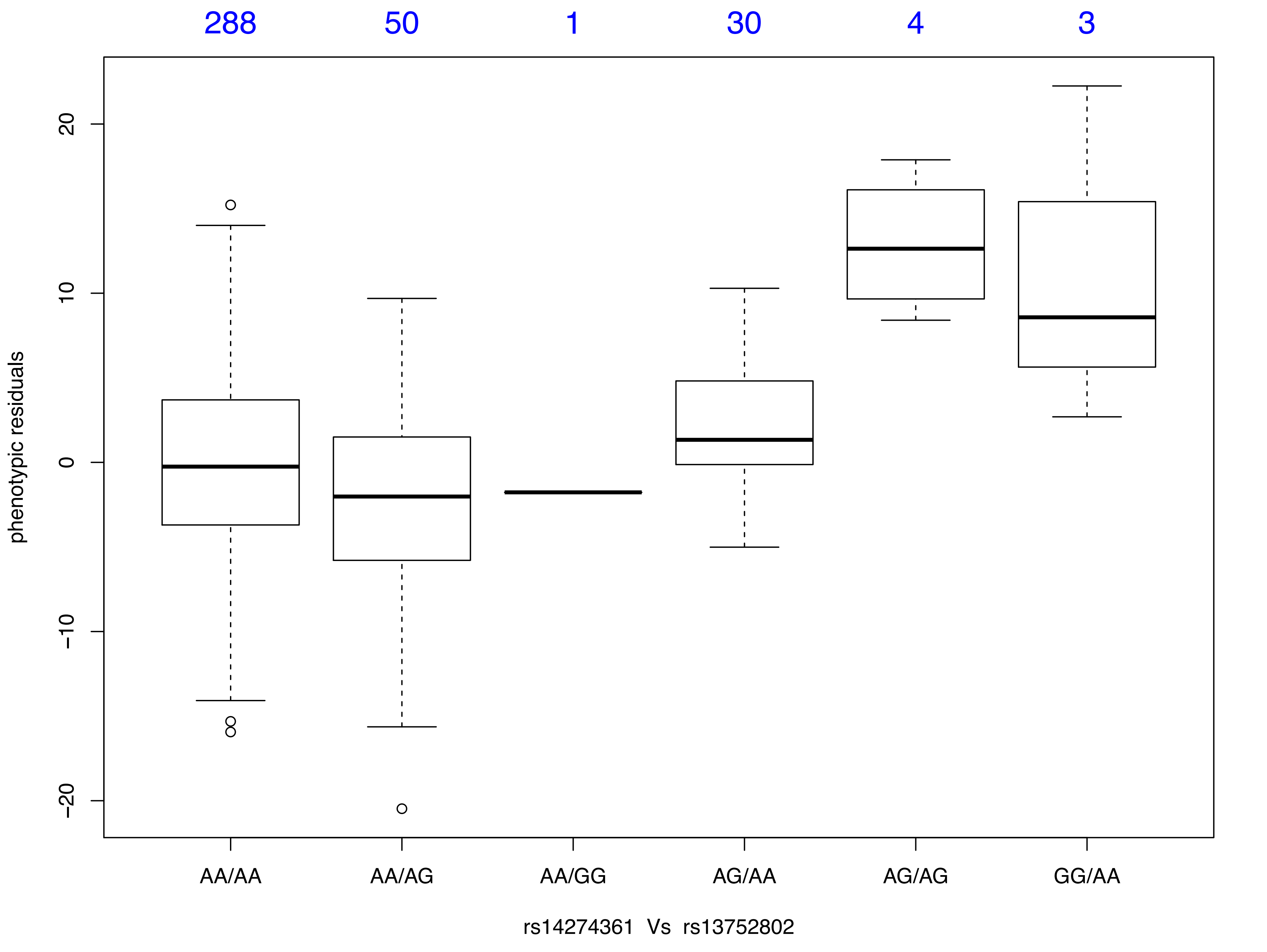
288
50
1
30
4
3
20
10
0
−10
−20
phenotypic residuals
AA/AA
AA/AG
AA/GG
AG/AA
AG/AG
GG/AA
rs14274361 Vs rs13752802

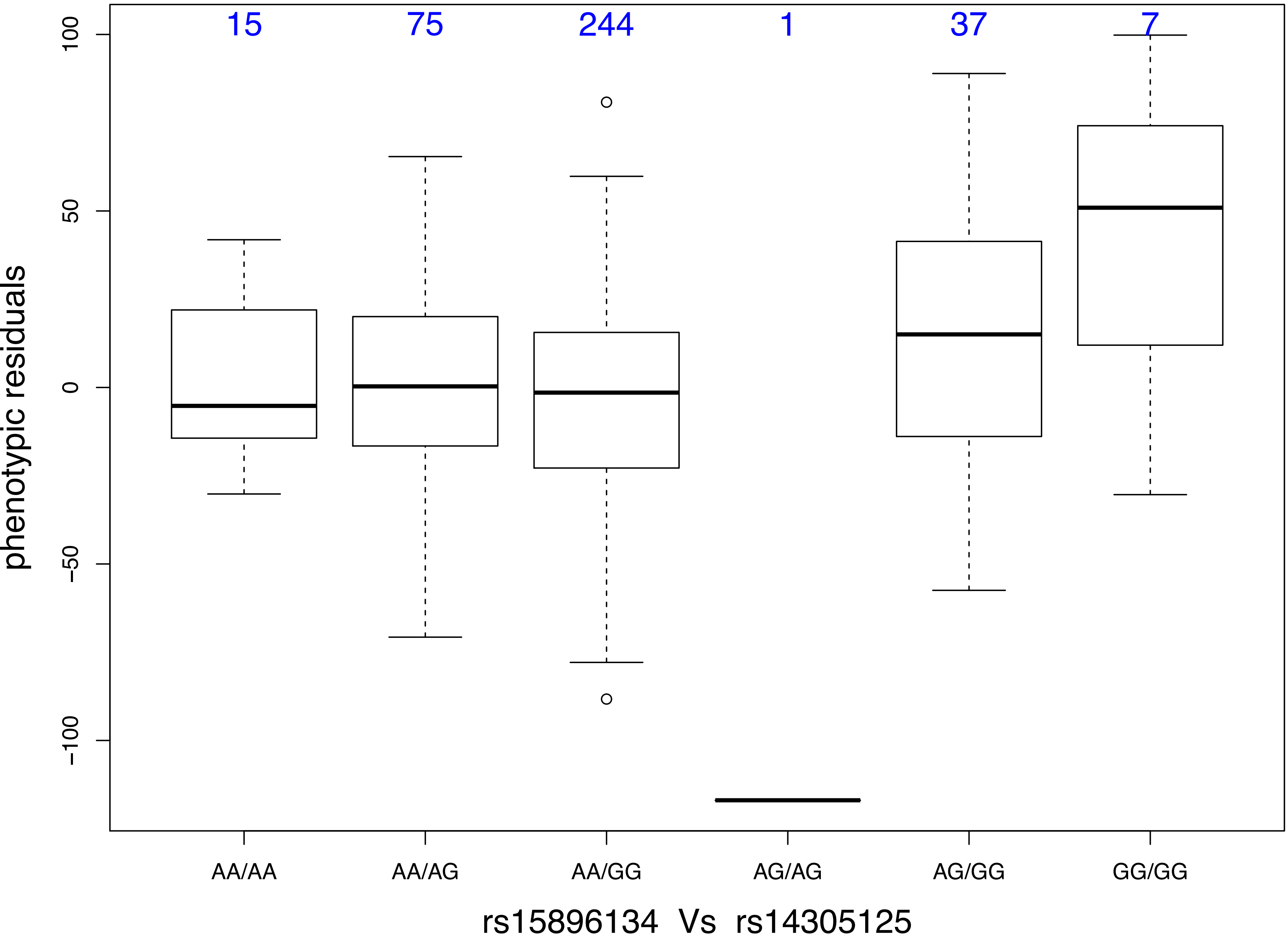

15
75
244
1
37
7
100
50
0
−50
−100
phenotypic residuals
AA/AA
AA/AG
AA/GG
AG/AG
AG/GG
GG/GG
rs15896134 Vs rs14305125

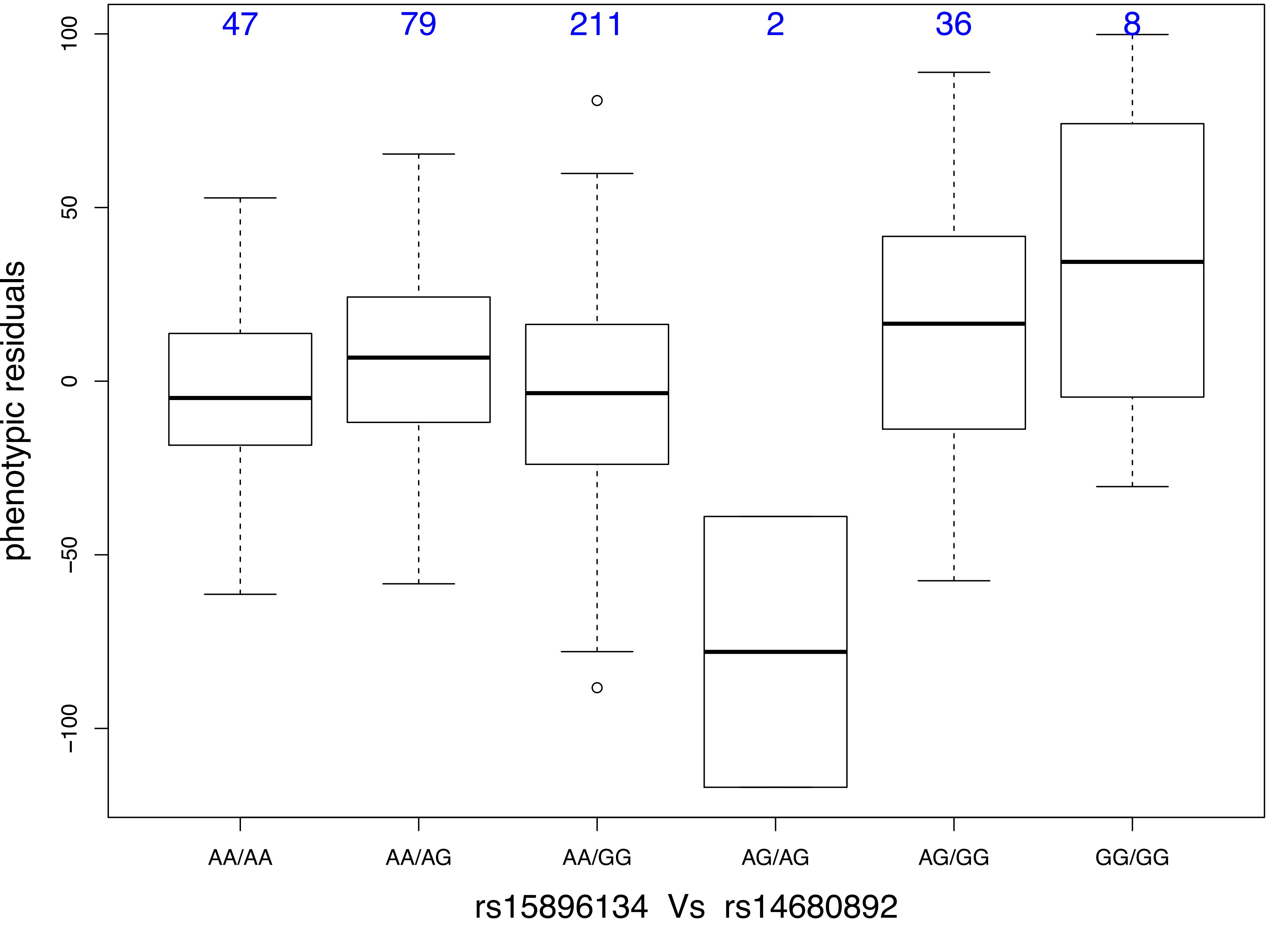
47
79
211
2
36
8
100
50
0
−50
−100
phenotypic residuals
AA/AA
AA/AG
AA/GG
AG/AG
AG/GG
GG/GG
rs15896134 Vs rs14680892

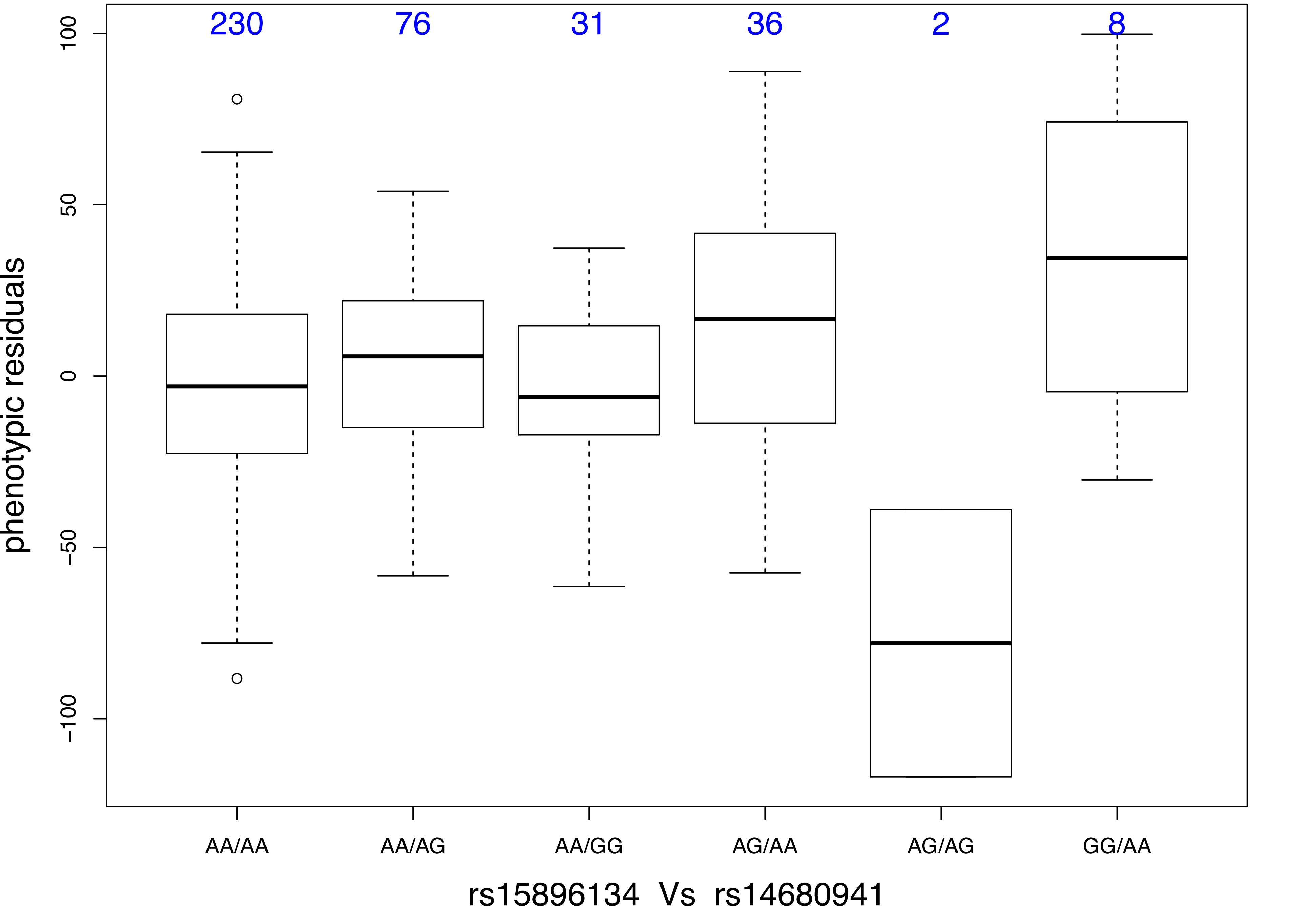
230
76
31
36
2
8
100
50
0
−50
−100
phenotypic residuals
AA/AA
AA/AG
AA/GG
AG/AA
AG/AG
GG/AA
rs15896134 Vs rs14680941

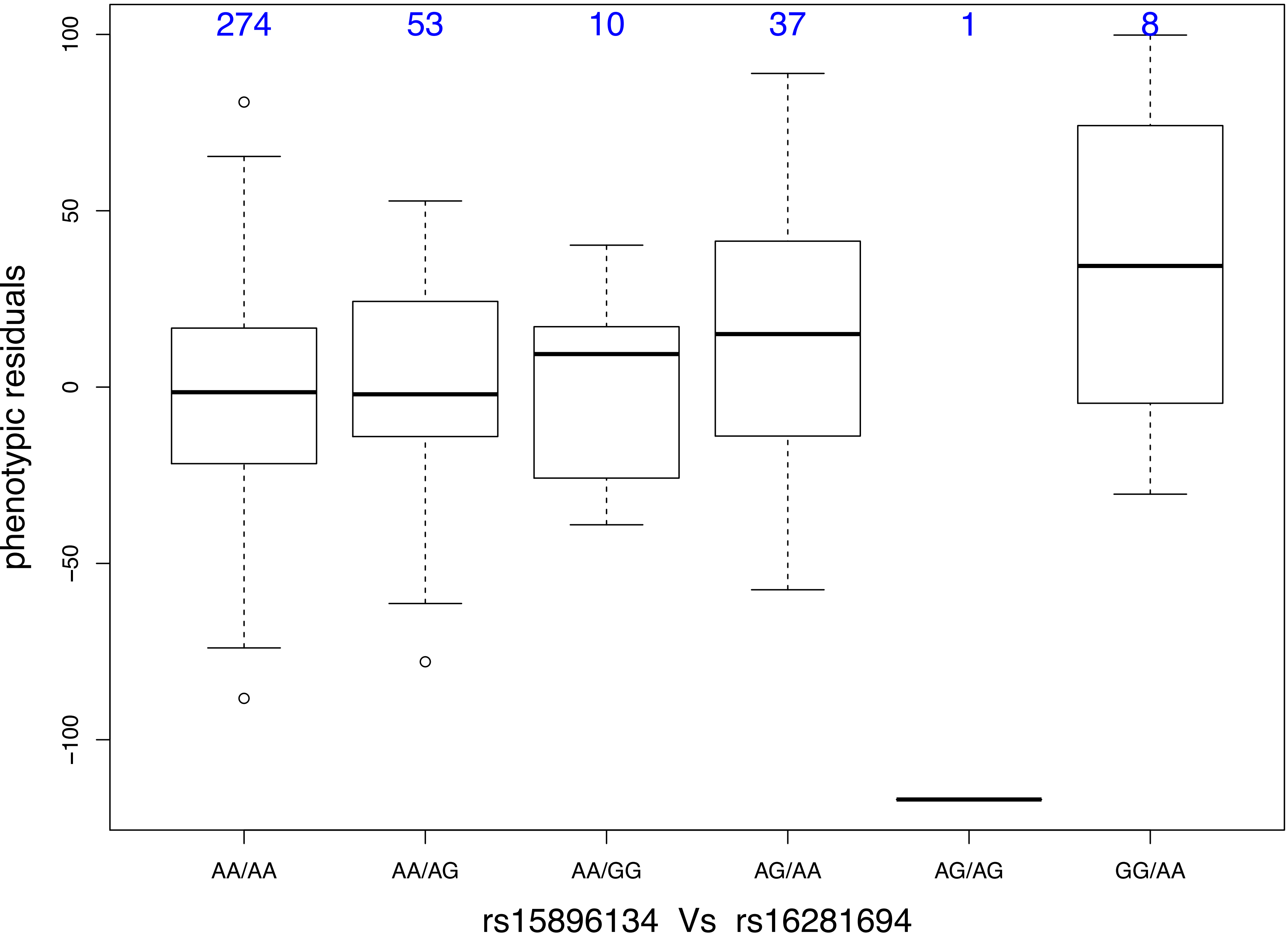

274
53
10
37
1
8
100
50
0
−50
−100
phenotypic residuals
AA/AA
AA/AG
AA/GG
AG/AA
AG/AG
GG/AA
rs15896134 Vs rs16281694

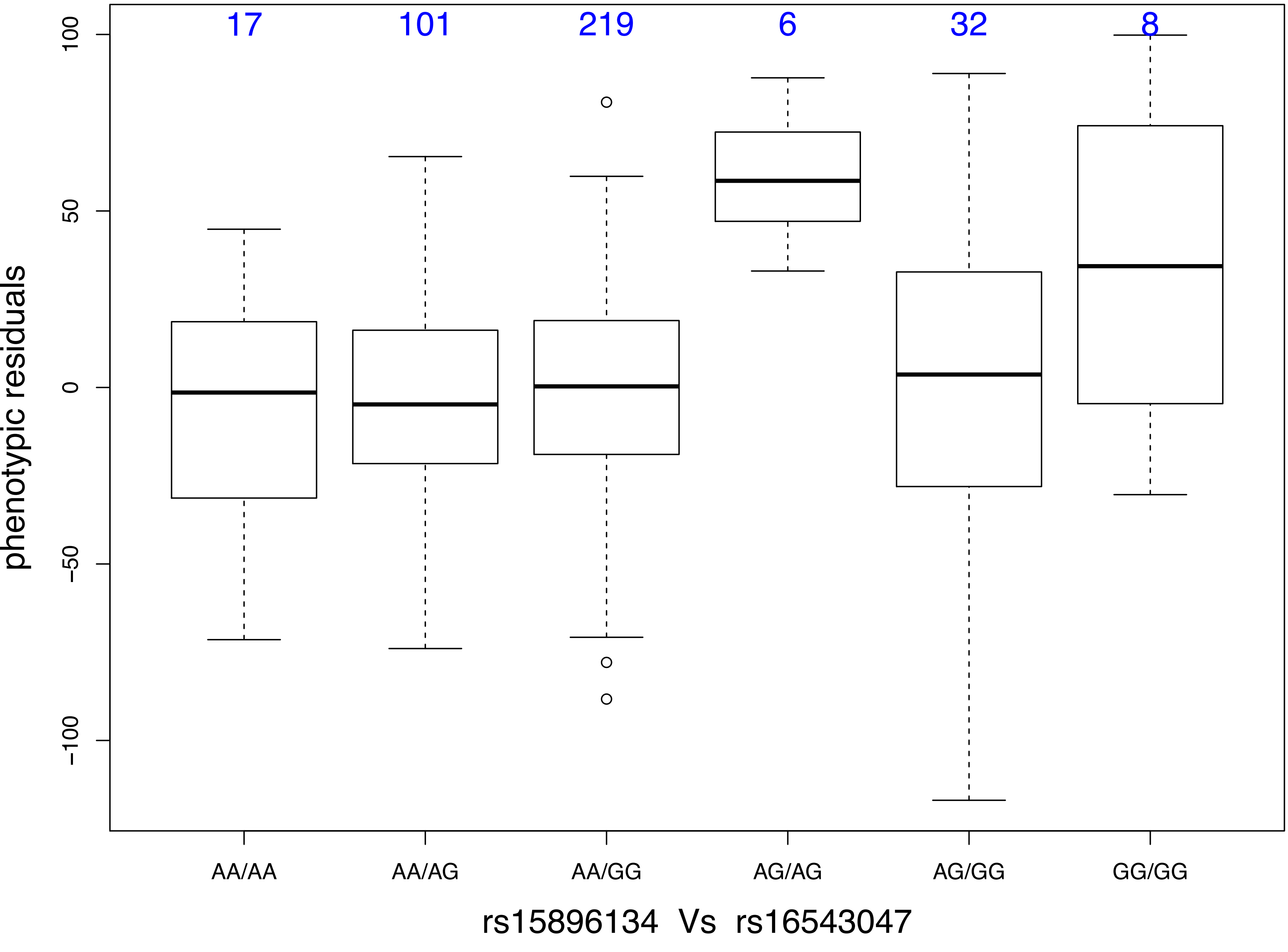

17
101
219
6
32
8
100
50
0
−50
−100
phenotypic residuals
AA/AA
AA/AG
AA/GG
AG/AG
AG/GG
GG/GG
rs15896134 Vs rs16543047

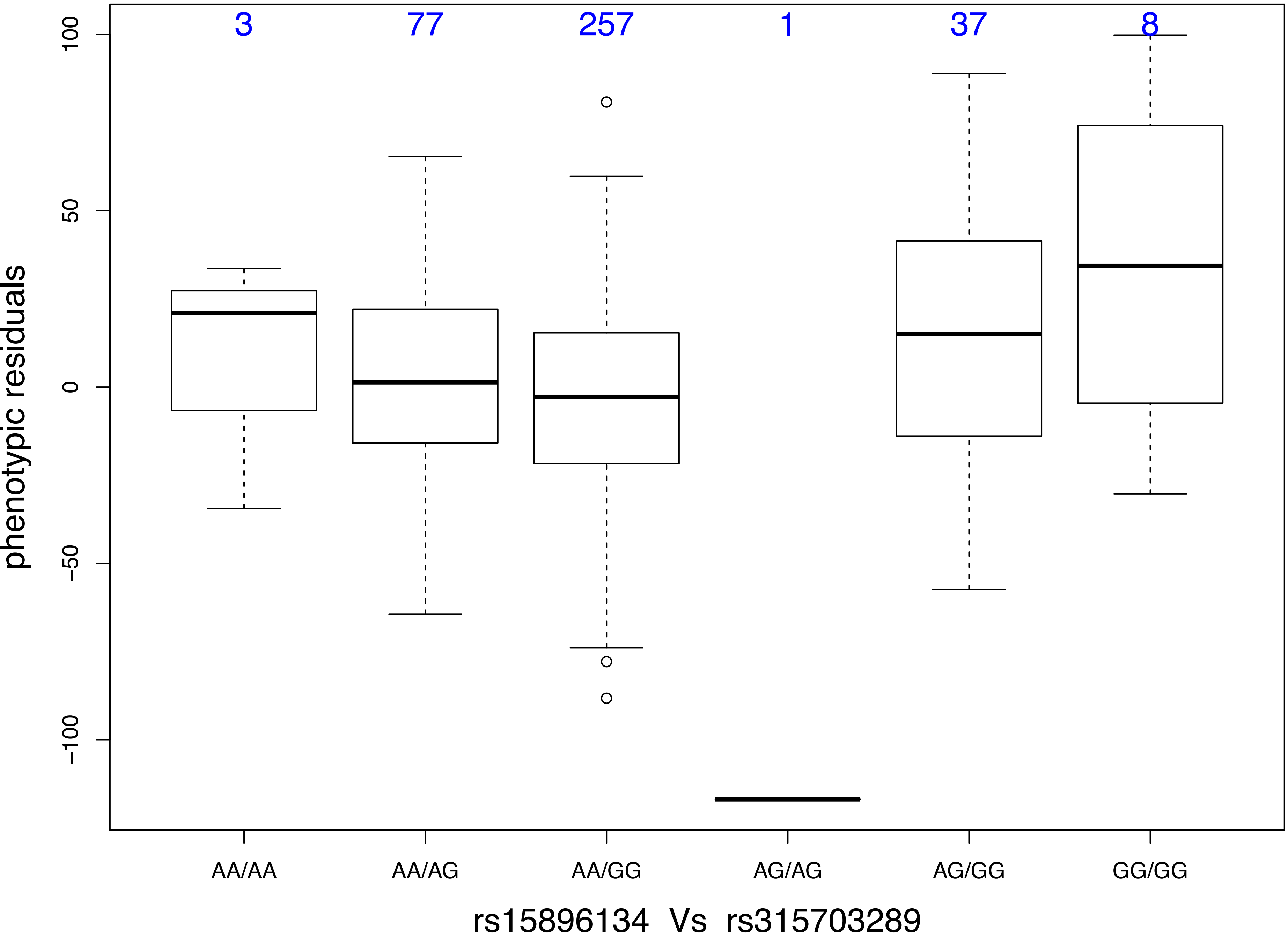
3
77
257
1
37
8
100
50
0
−50
−100
phenotypic residuals
AA/AA
AA/AG
AA/GG
AG/AG
AG/GG
GG/GG
rs15896134 Vs rs315703289

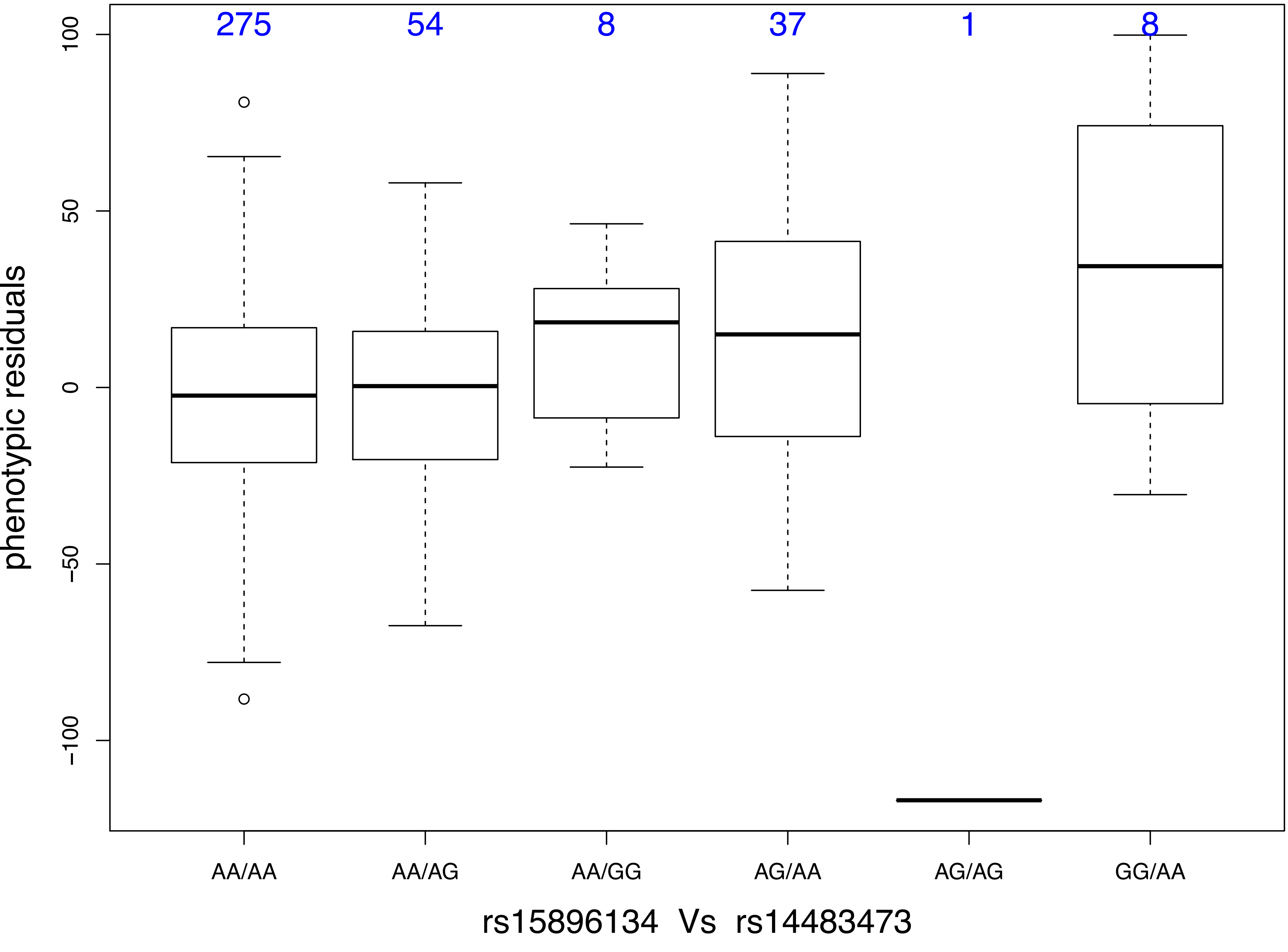

275
54
8
37
1
8
100
50
0
−50
−100
phenotypic residuals
AA/AA
AA/AG
AA/GG
AG/AA
AG/AG
GG/AA
rs15896134 Vs rs14483473

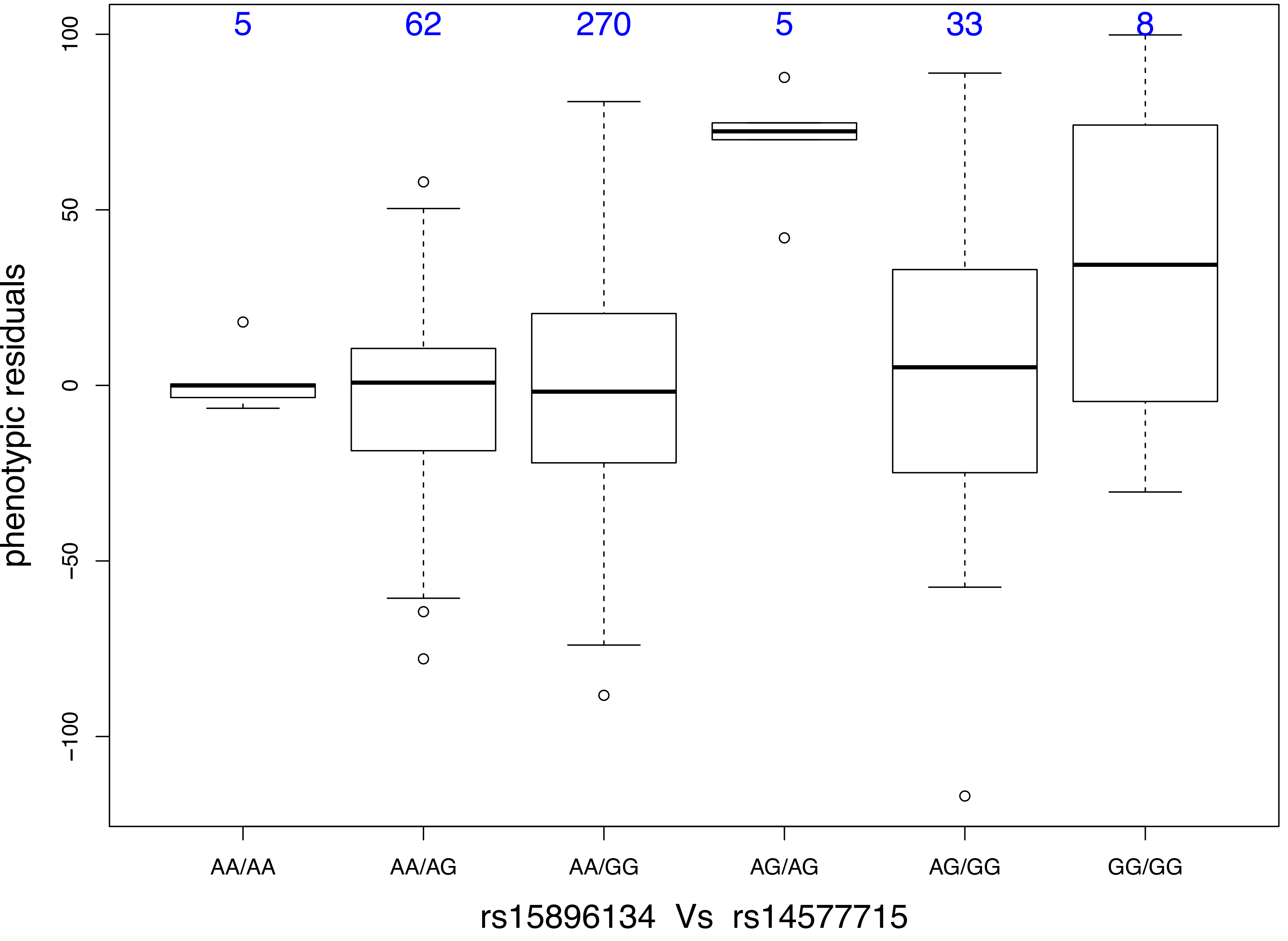

5
62
270
5
33
8
100
50
0
−50
−100
phenotypic residuals
AA/AA
AA/AG
AA/GG
AG/AG
AG/GG
GG/GG
rs15896134 Vs rs14577715

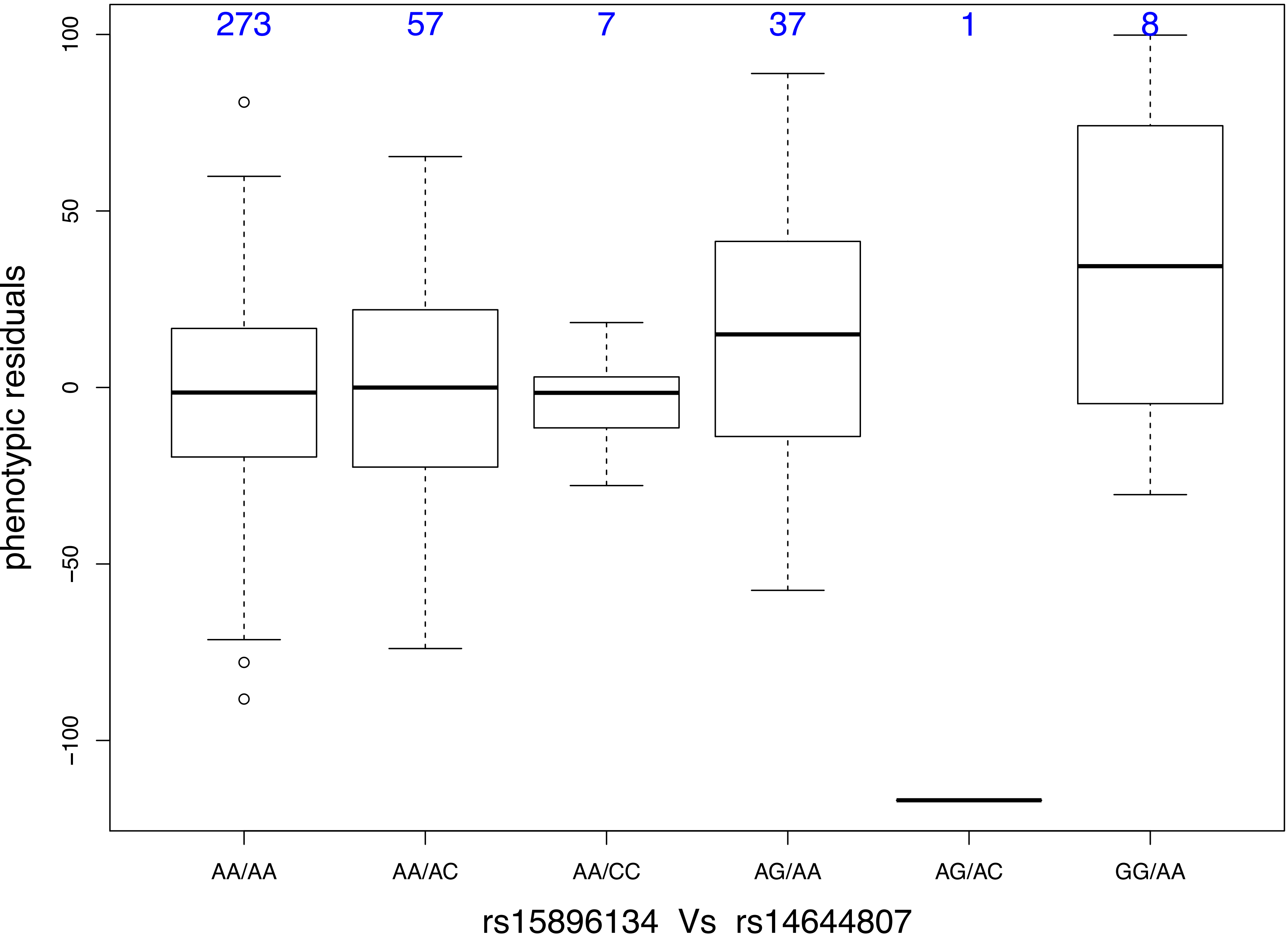
273
57
7
37
1
8
100
50
0
−50
−100
phenotypic residuals
AA/AA
AA/AC
AA/CC
AG/AA
AG/AC
GG/AA
rs15896134 Vs rs14644807

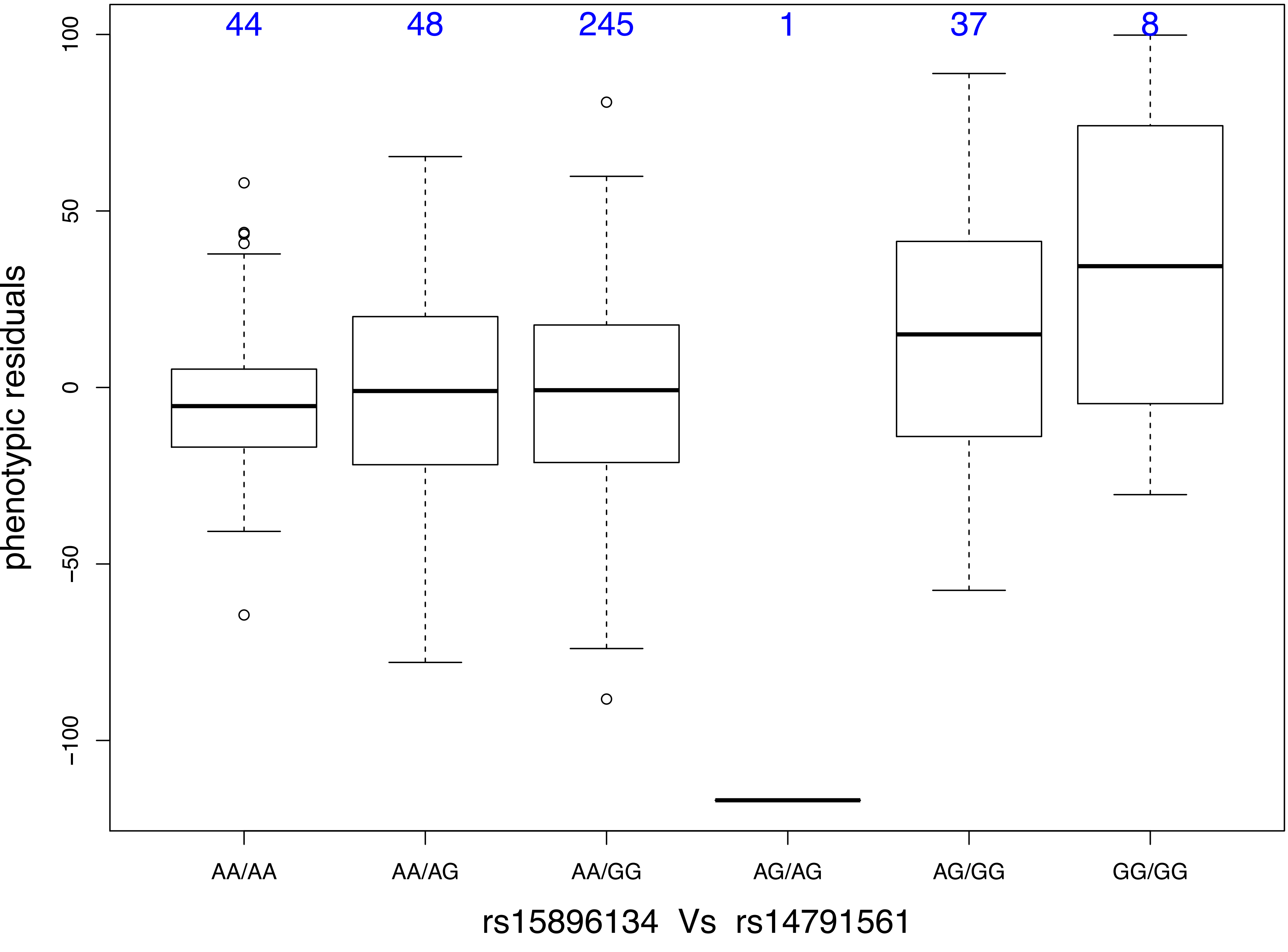
44
48
245
1
37
8
100
50
0
−50
−100
phenotypic residuals
AA/AA
AA/AG
AA/GG
AG/AG
AG/GG
GG/GG
rs15896134 Vs rs14791561

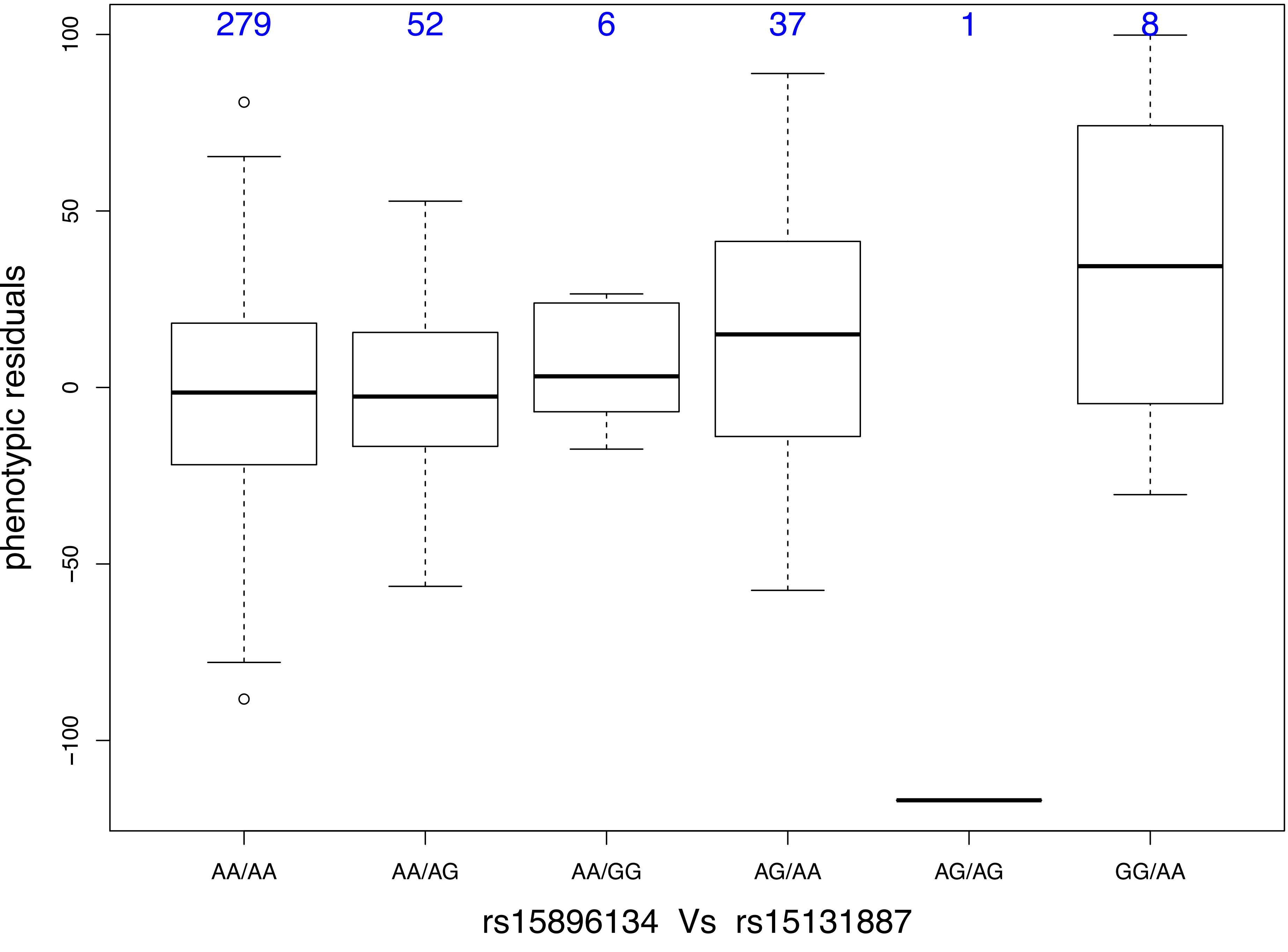

279
52
6
37
1
8
100
50
0
−50
−100
phenotypic residuals
AA/AA
AA/AG
AA/GG
AG/AA
AG/AG
GG/AA
rs15896134 Vs rs15131887

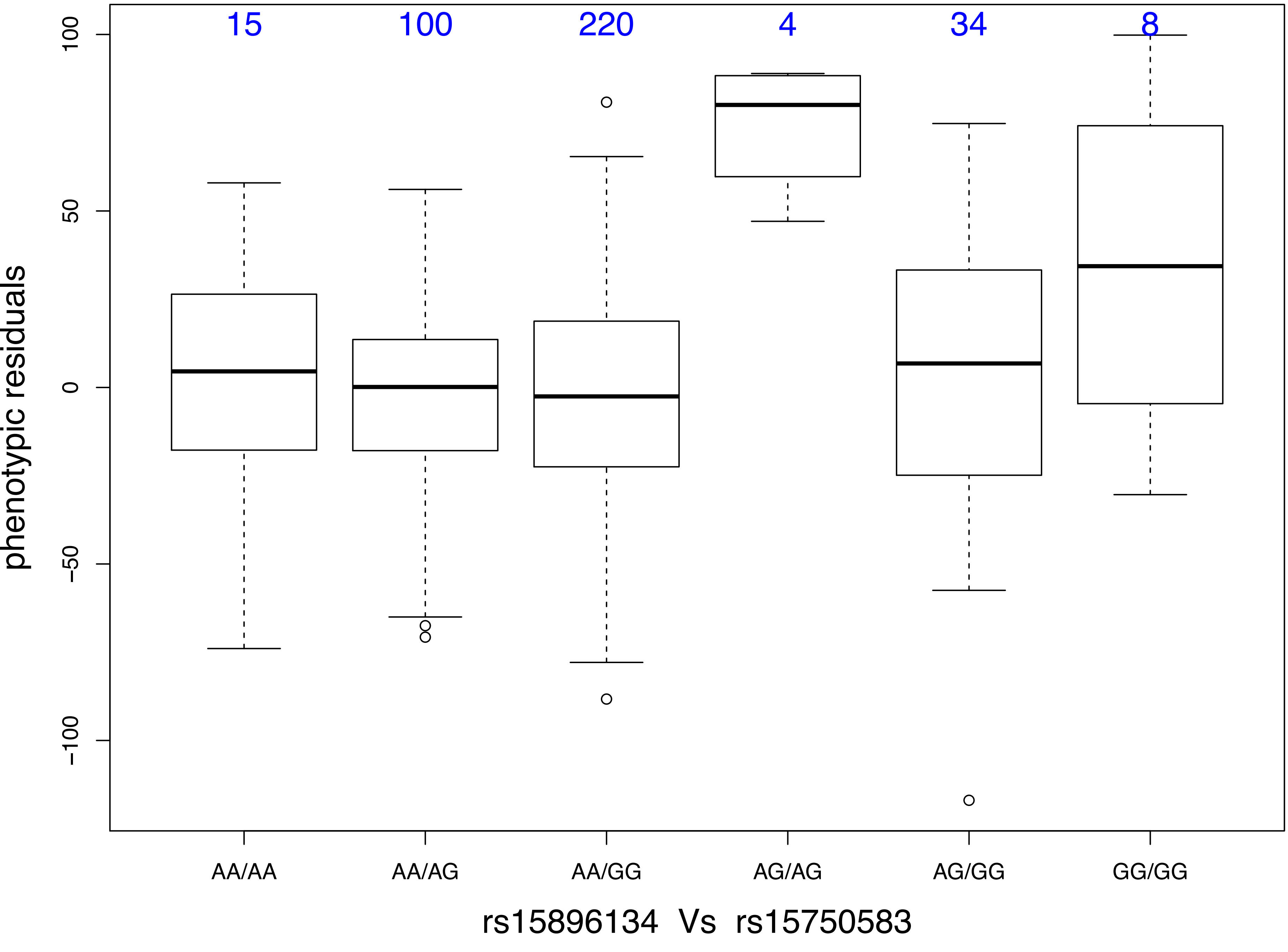

15
100
220
4
34
8
100
50
0
−50
−100
phenotypic residuals
AA/AA
AA/AG
AA/GG
AG/AG
AG/GG
GG/GG
rs15896134 Vs rs15750583

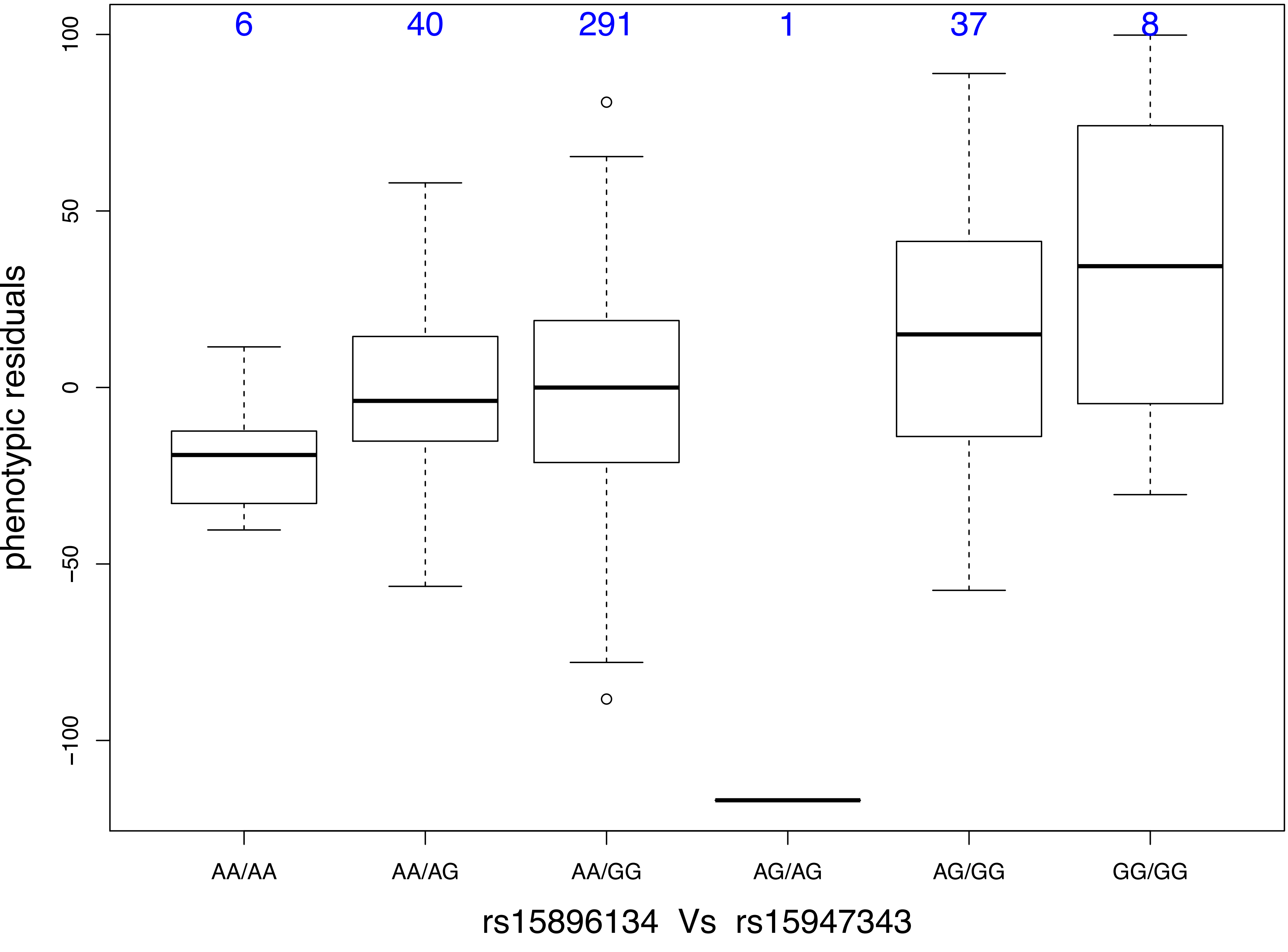

6
40
291
1
37
8
100
50
0
−50
−100
phenotypic residuals
AA/AA
AA/AG
AA/GG
AG/AG
AG/GG
GG/GG
rs15896134 Vs rs15947343

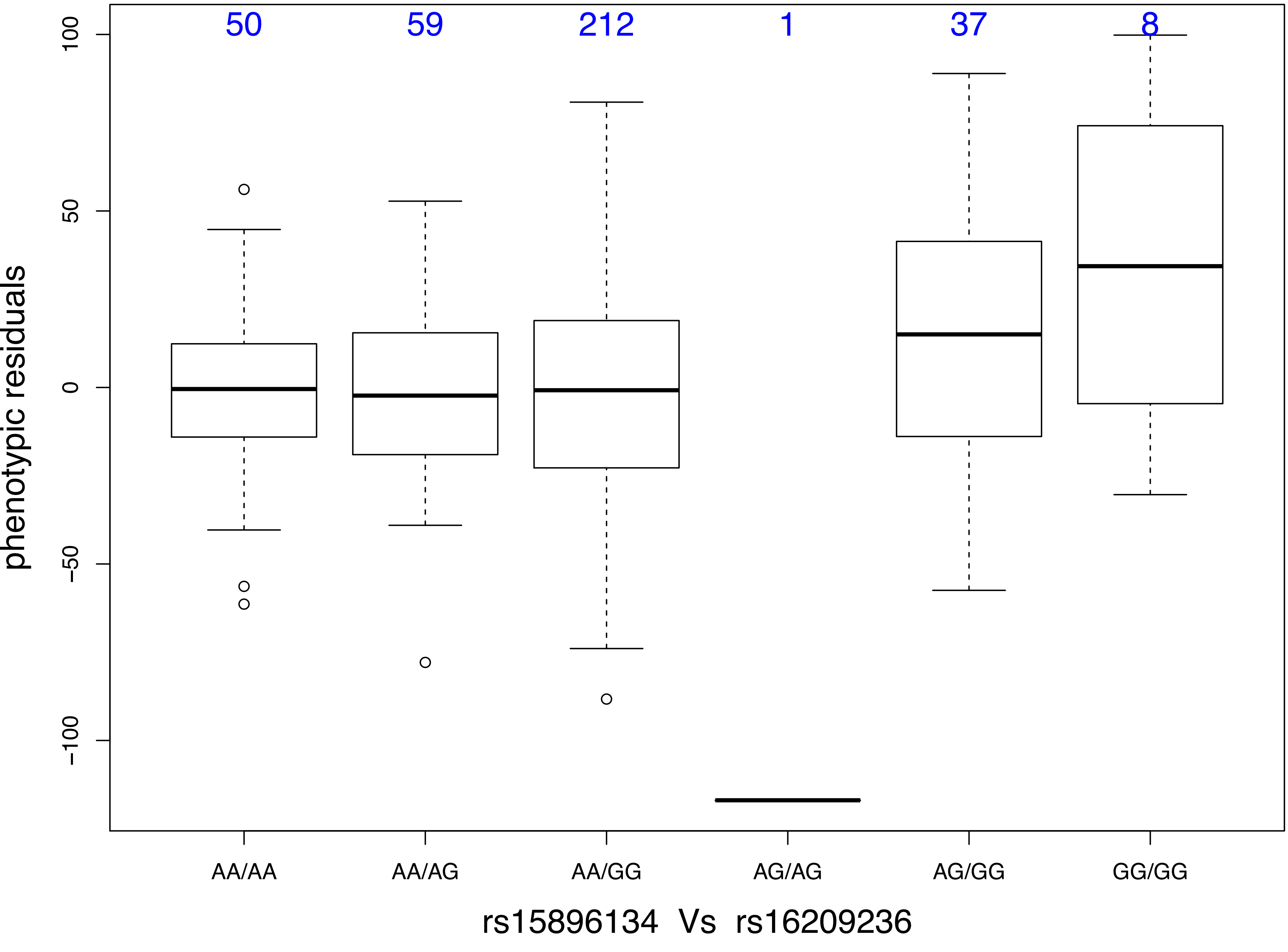
50
59
212
1
37
8
100
50
0
−50
−100
phenotypic residuals
AA/AA
AA/AG
AA/GG
AG/AG
AG/GG
GG/GG
rs15896134 Vs rs16209236

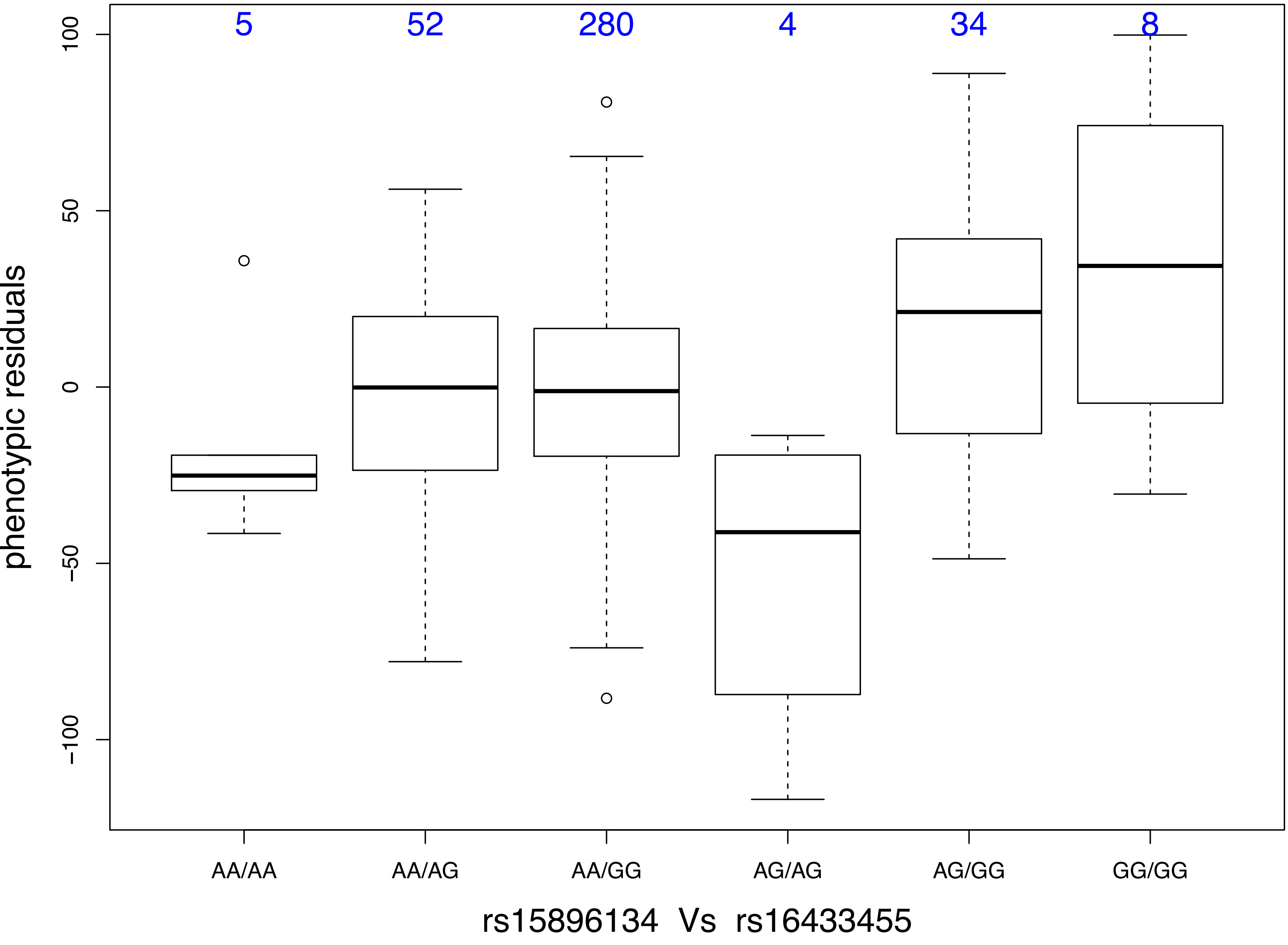
5
52
280
4
34
8
100
50
0
−50
−100
phenotypic residuals
AA/AA
AA/AG
AA/GG
AG/AG
AG/GG
GG/GG
rs15896134 Vs rs16433455

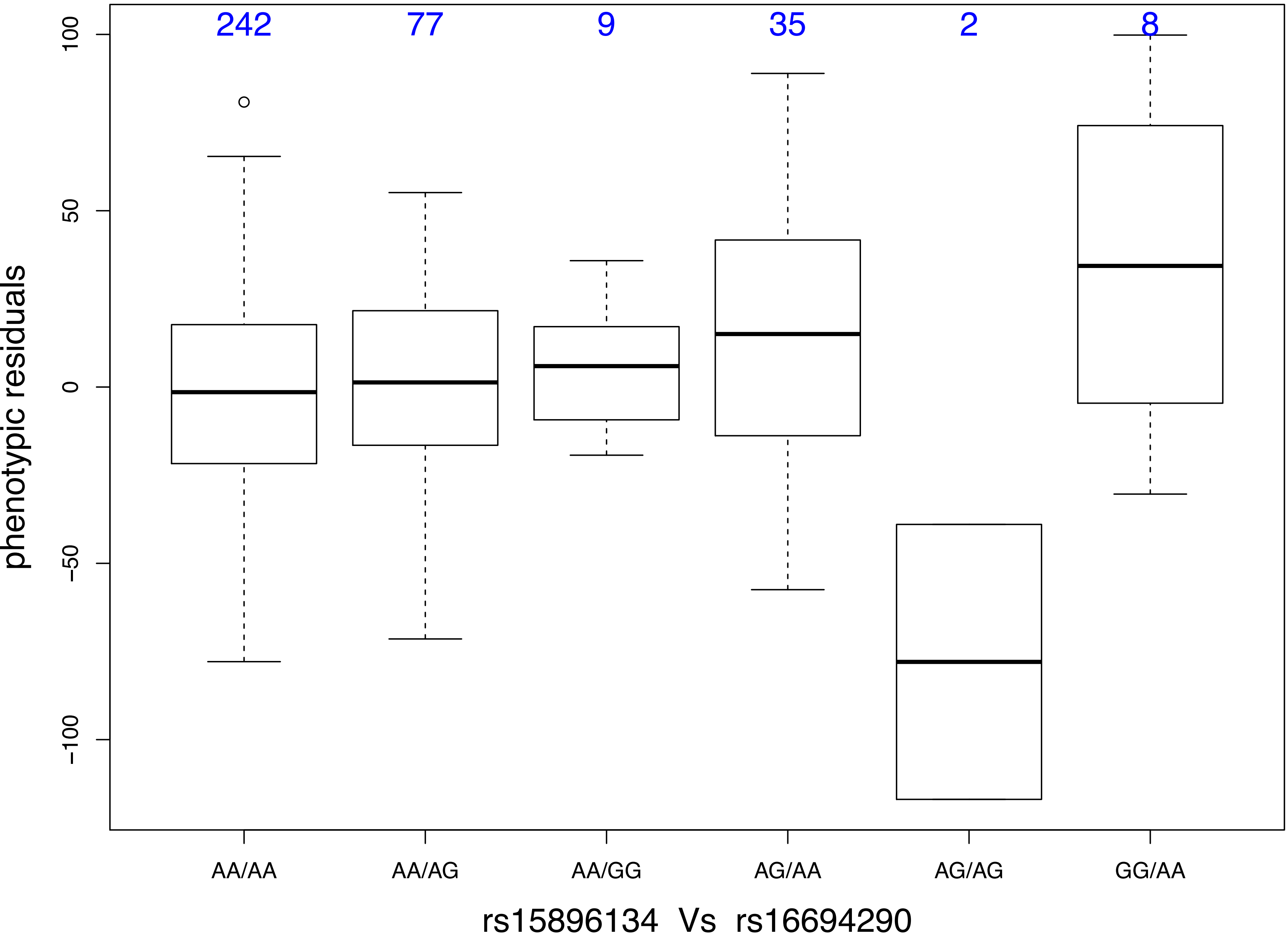

242
77
9
35
2
8
100
50
0
−50
−100
phenotypic residuals
AA/AA
AA/AG
AA/GG
AG/AA
AG/AG
GG/AA
rs15896134 Vs rs16694290

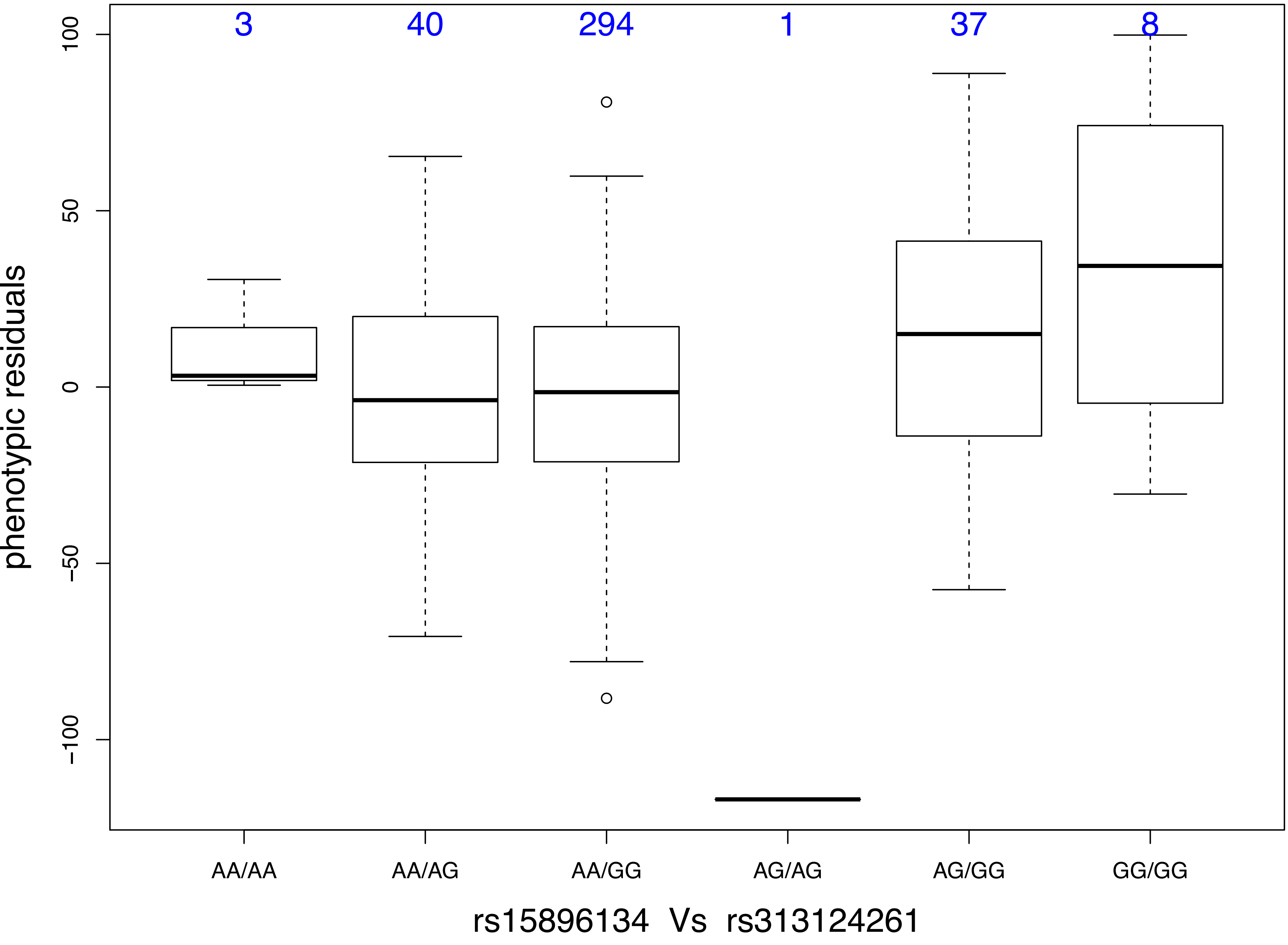
3
40
294
1
37
8
100
50
0
−50
−100
phenotypic residuals
AA/AA
AA/AG
AA/GG
AG/AG
AG/GG
GG/GG
rs15896134 Vs rs313124261

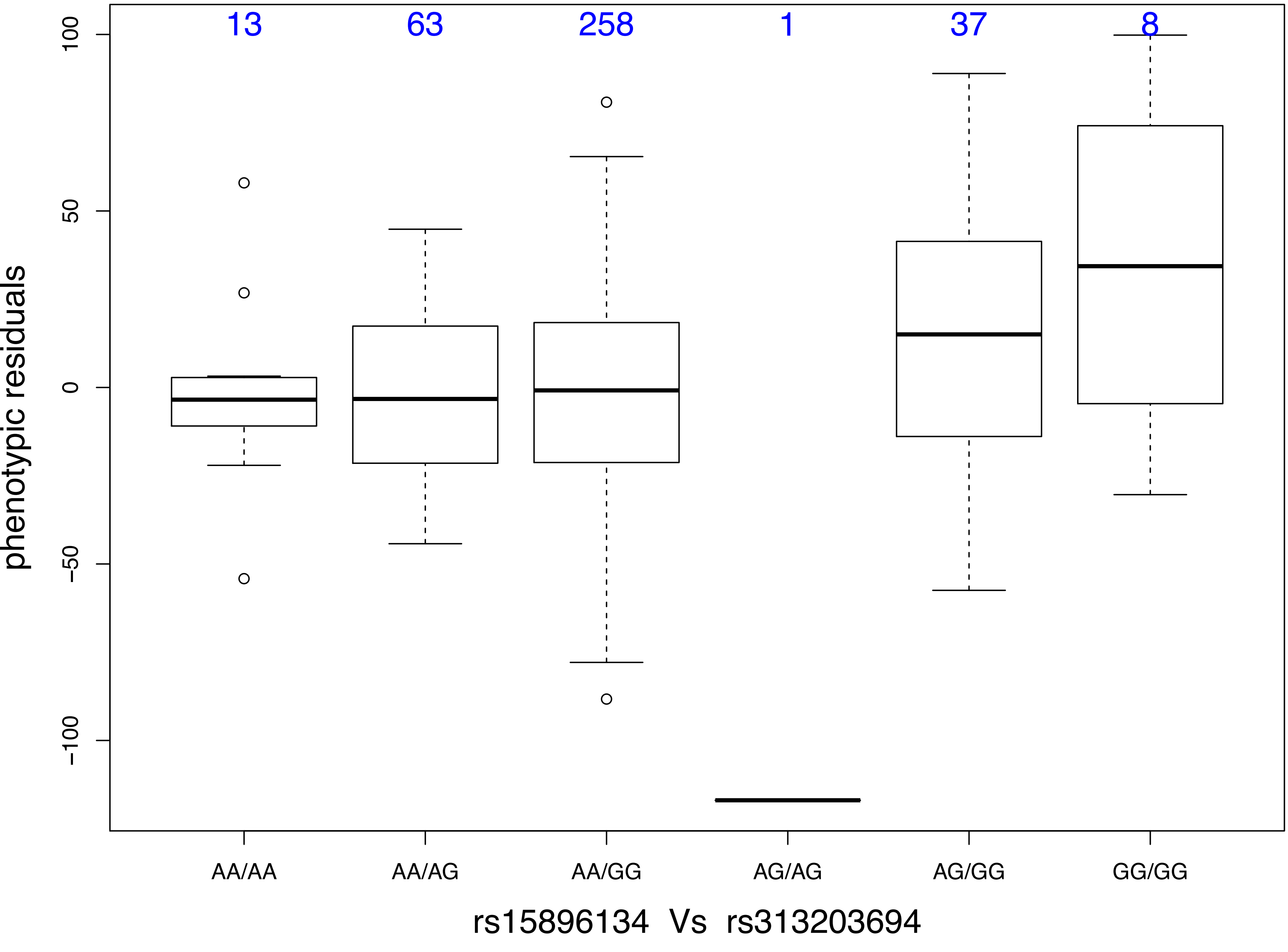

13
63
258
1
37
8
100
50
0
−50
−100
phenotypic residuals
AA/AA
AA/AG
AA/GG
AG/AG
AG/GG
GG/GG
rs15896134 Vs rs313203694

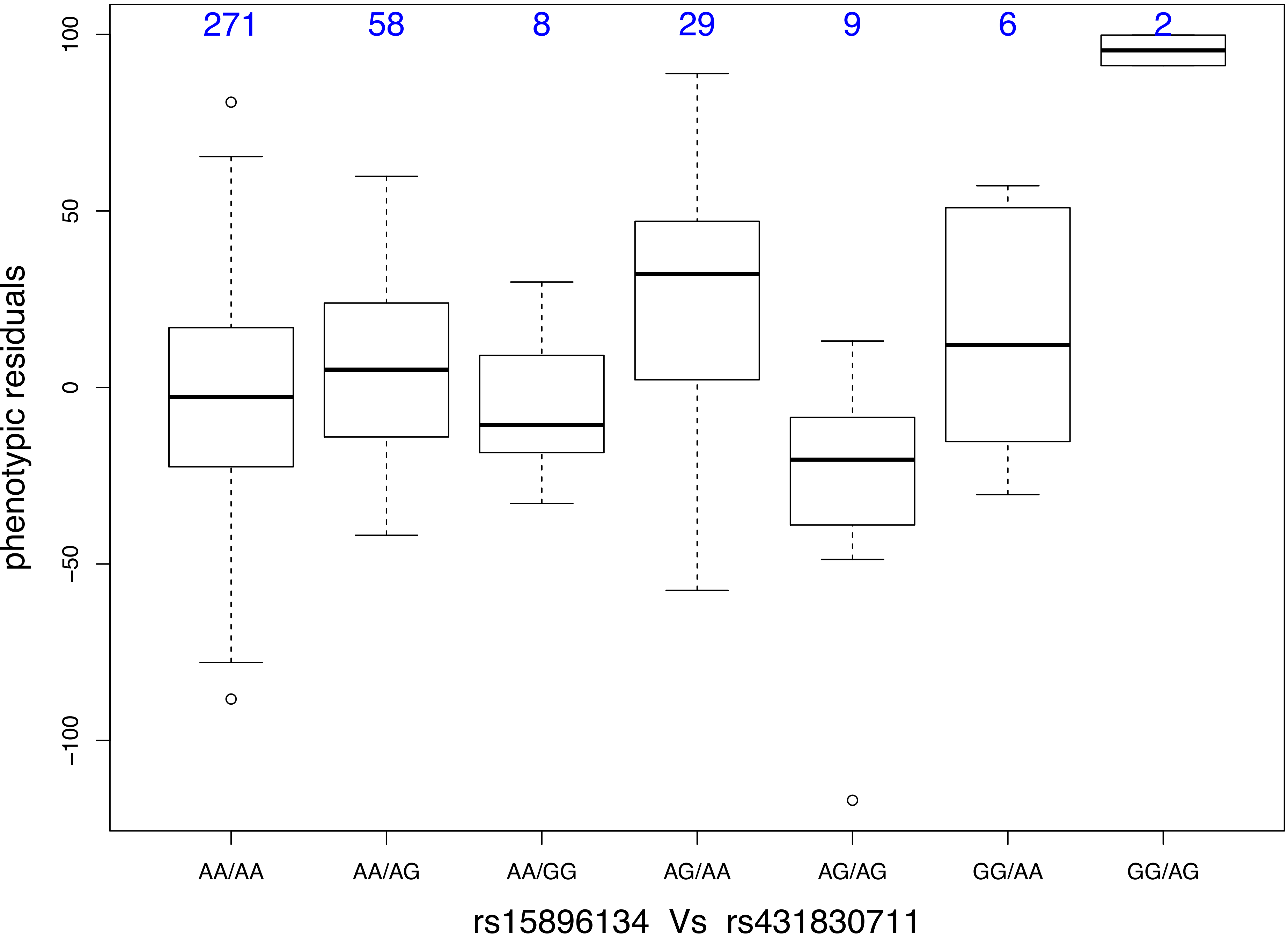

271
58
8
29
9
6
2
100
50
0
−50
−100
phenotypic residuals
AA/AA
AA/AG
AA/GG
AG/AA
AG/AG
GG/AA
GG/AG
rs15896134 Vs rs431830711

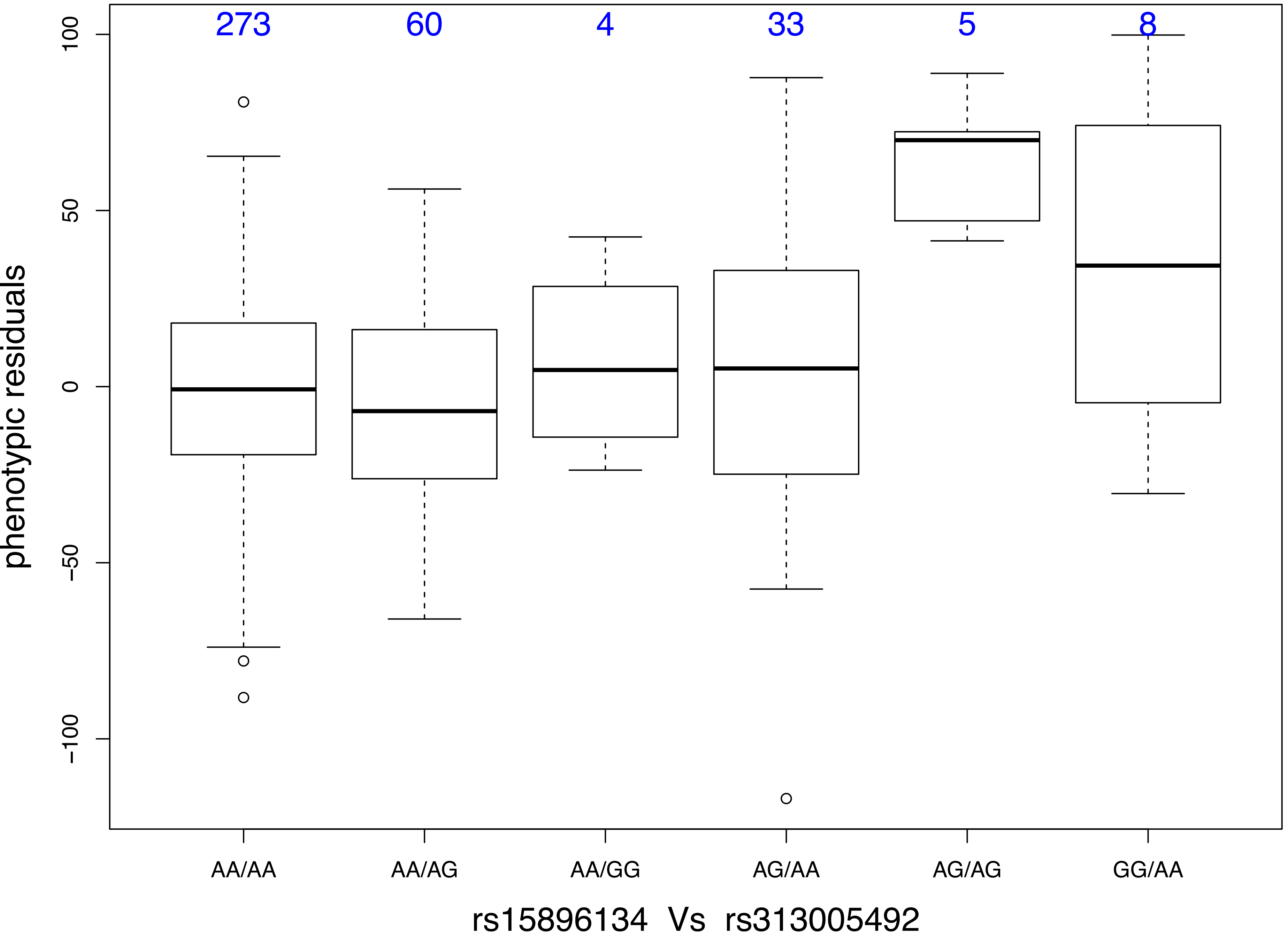
273
60
4
33
5
8
100
50
0
−50
−100
phenotypic residuals
AA/AA
AA/AG
AA/GG
AG/AA
AG/AG
GG/AA
rs15896134 Vs rs313005492

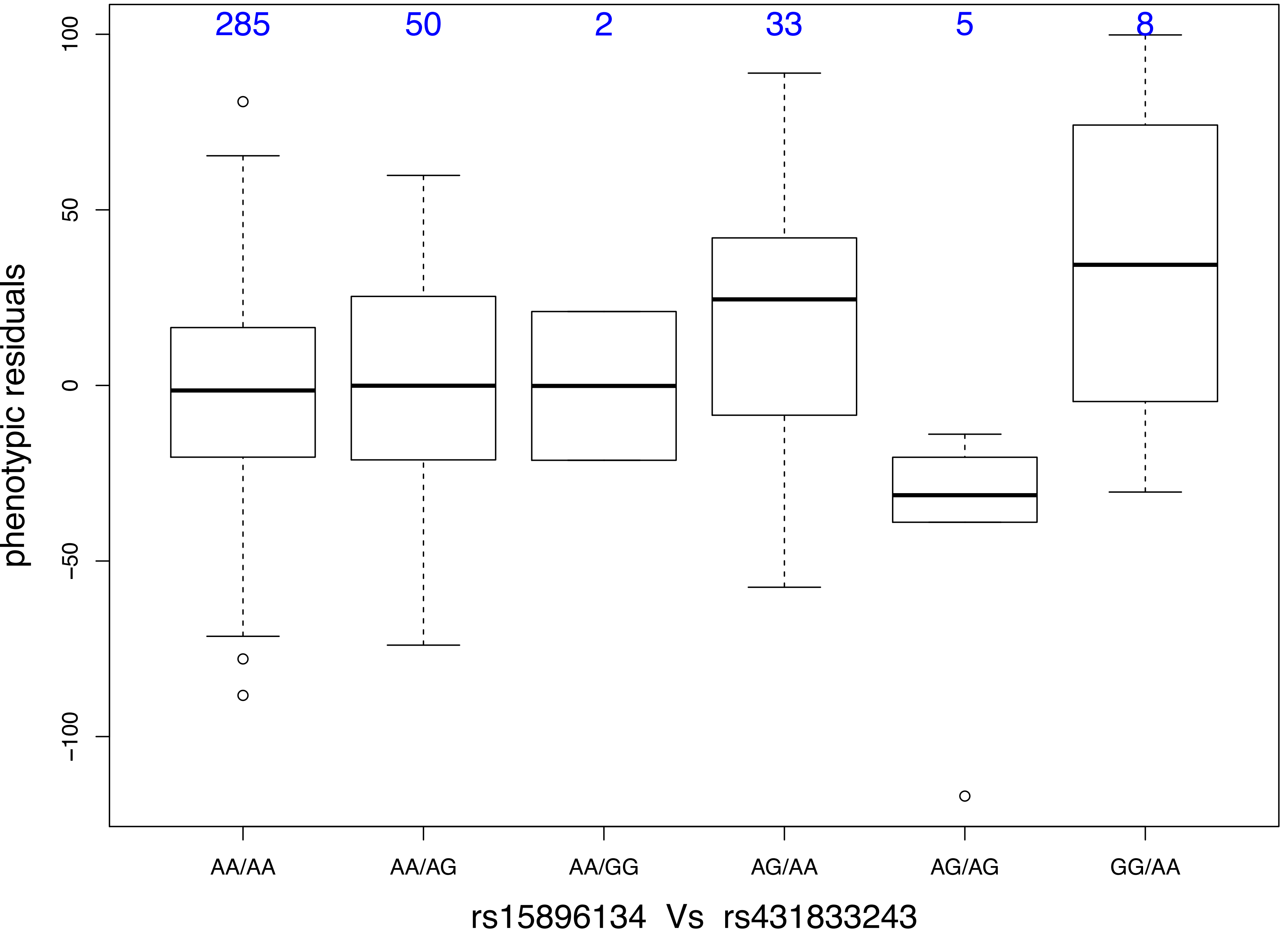

285
50
2
33
5
8
100
50
0
−50
−100
phenotypic residuals
AA/AA
AA/AG
AA/GG
AG/AA
AG/AG
GG/AA
rs15896134 Vs rs431833243

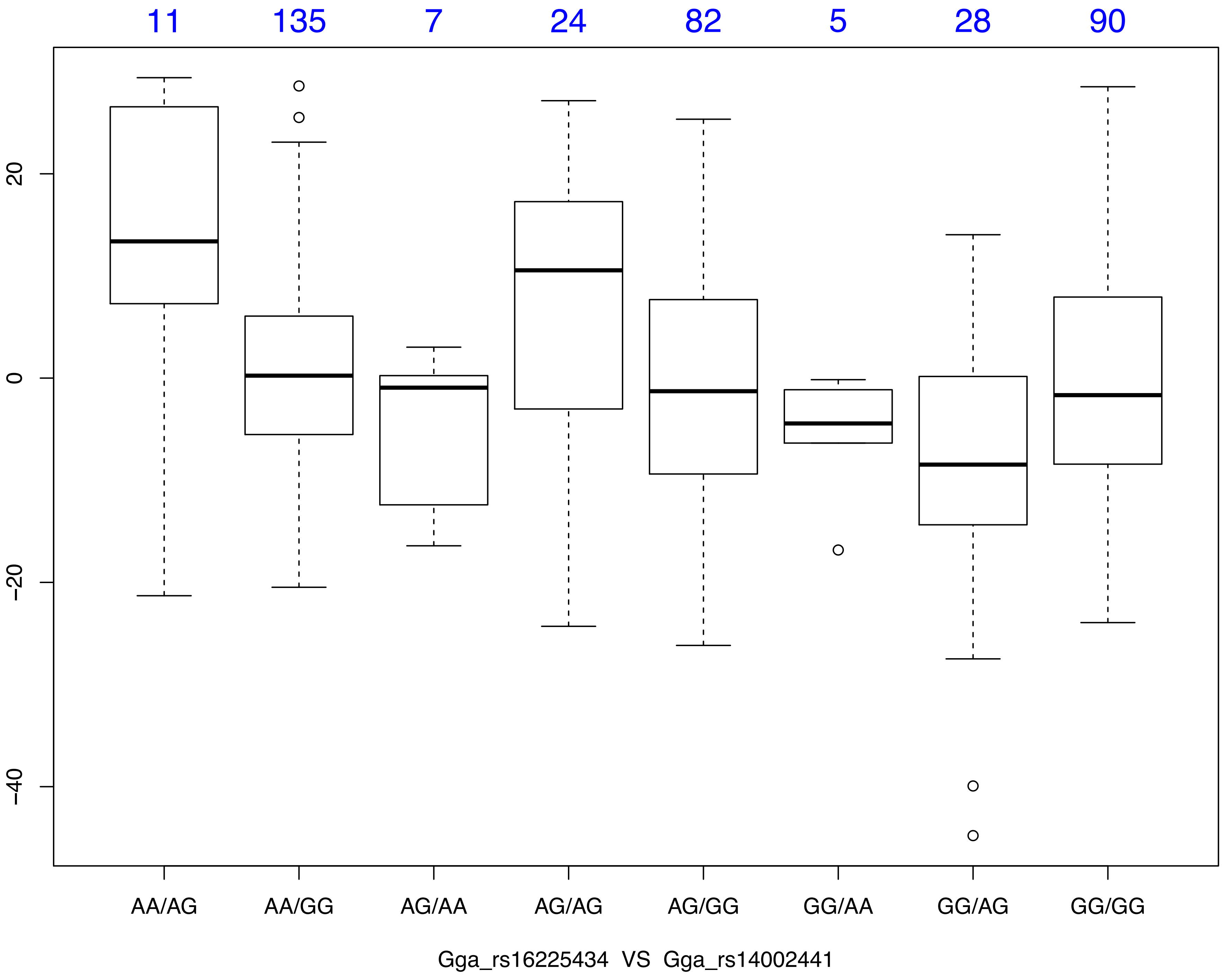
11
135
7
24
82
5
28
90
20
0
−20
−40
AA/AG
AA/GG
AG/AA
AG/AG
AG/GG
GG/AA
GG/AG
GG/GG
Gga_rs16225434 VS Gga_rs14002441

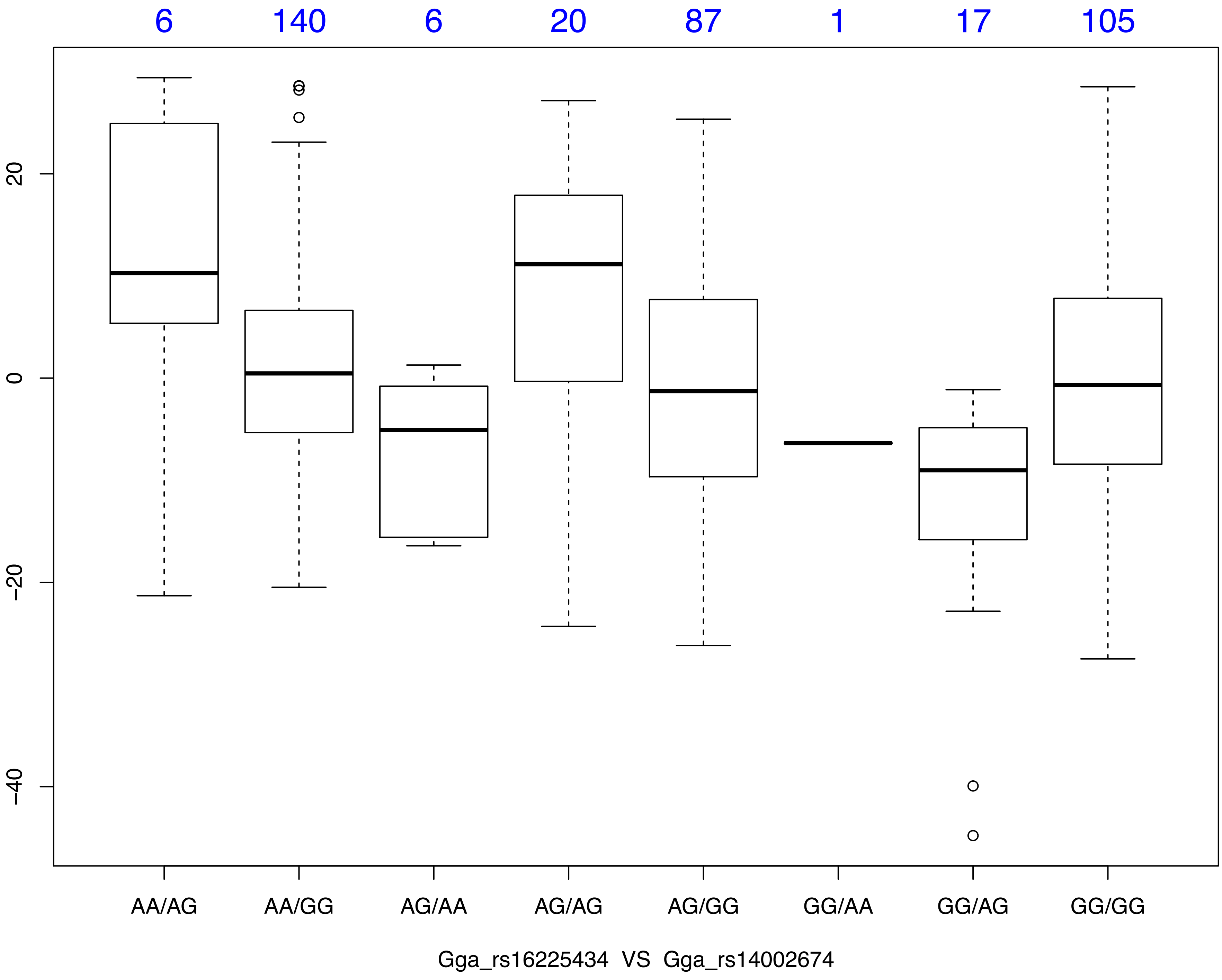

6
140
6
20
87
1
17
105
20
0
−20
−40
AA/AG
AA/GG
AG/AA
AG/AG
AG/GG
GG/AA
GG/AG
GG/GG
Gga_rs16225434 VS Gga_rs14002674

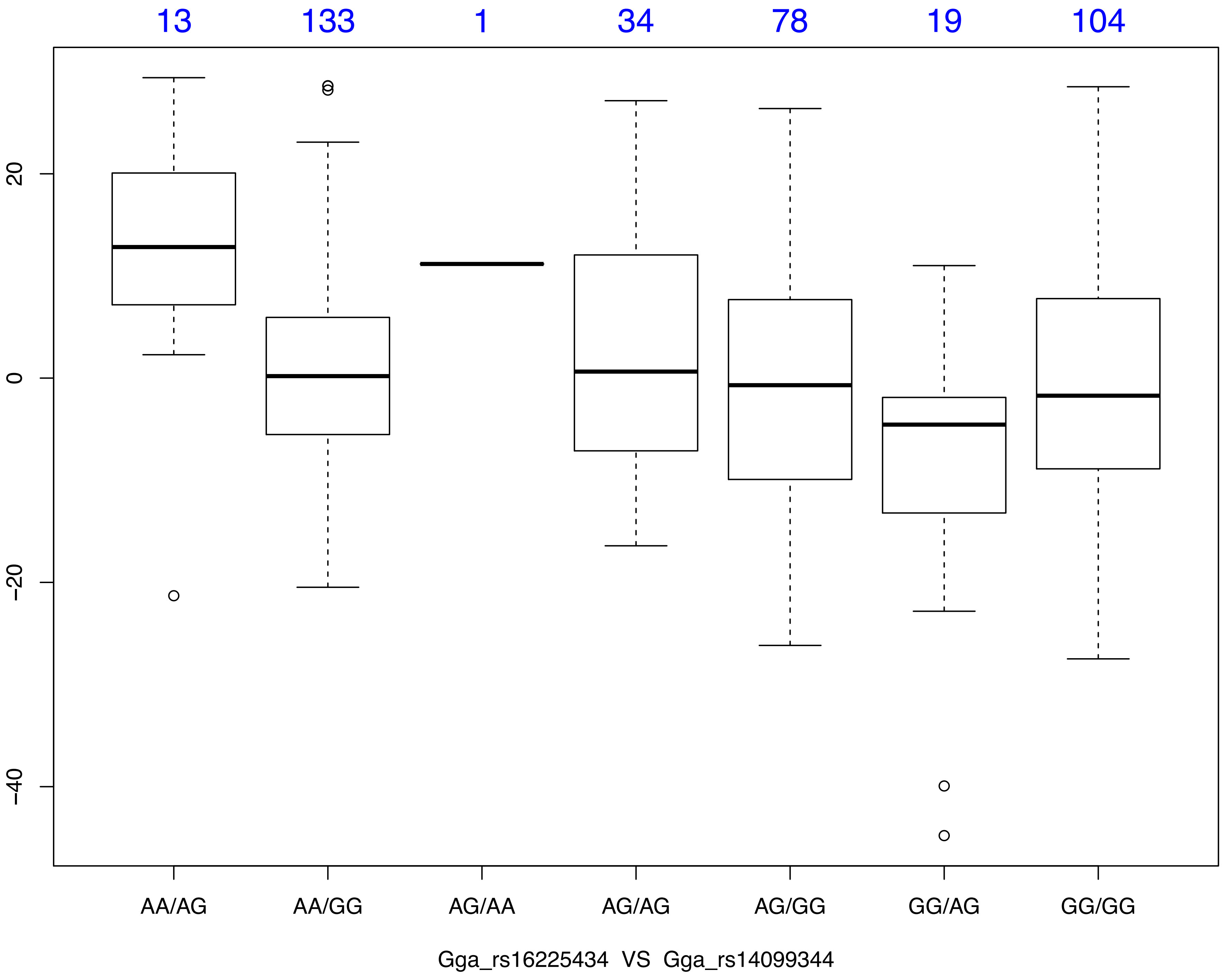

13
133
1
34
78
19
104
20
0
−20
−40
AA/AG
AA/GG
AG/AA
AG/AG
AG/GG
GG/AG
GG/GG
Gga_rs16225434 VS Gga_rs14099344

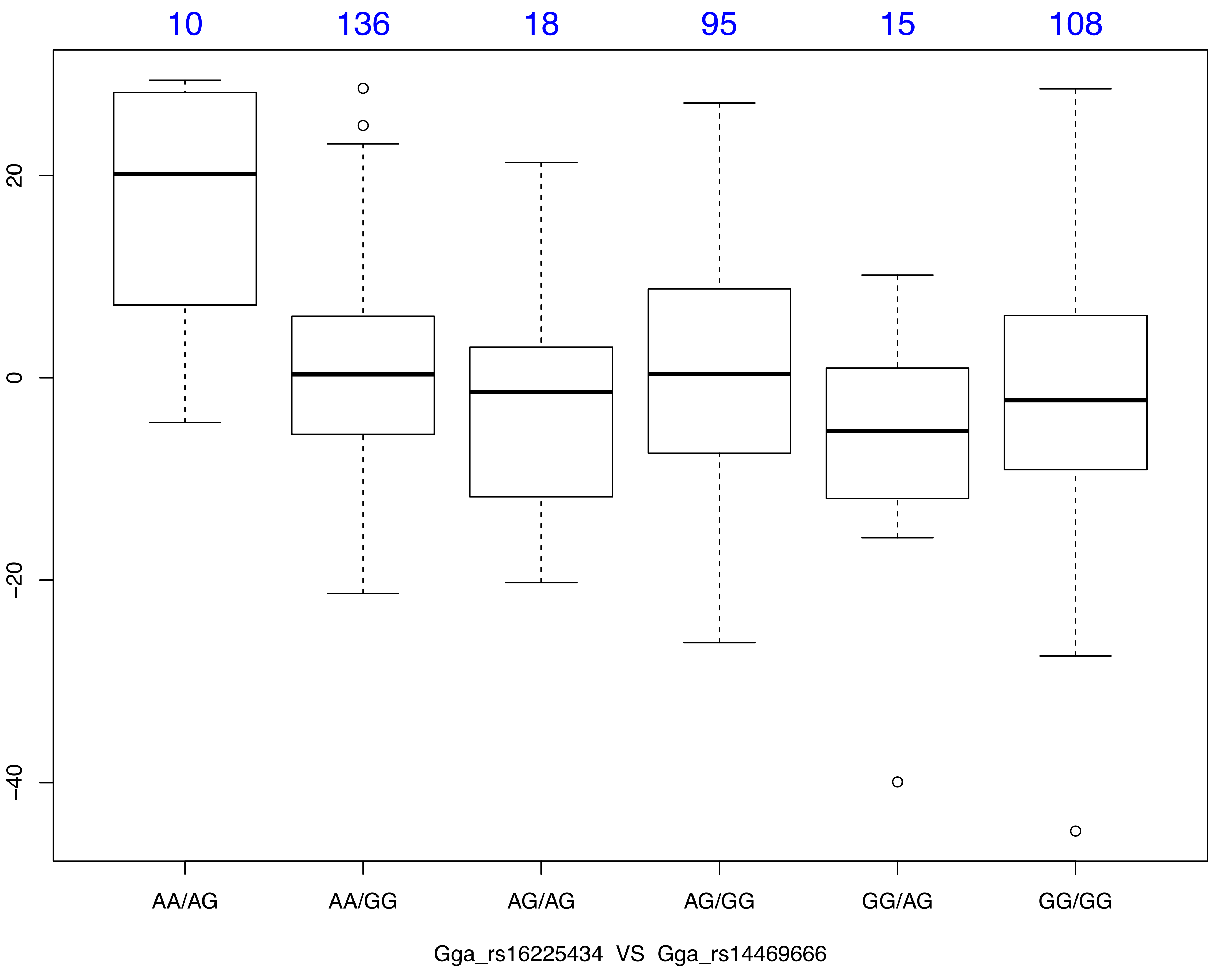

10
136
18
95
15
108
20
0
−20
−40
AA/AG
AA/GG
AG/AG
AG/GG
GG/AG
GG/GG
Gga_rs16225434 VS Gga_rs14469666

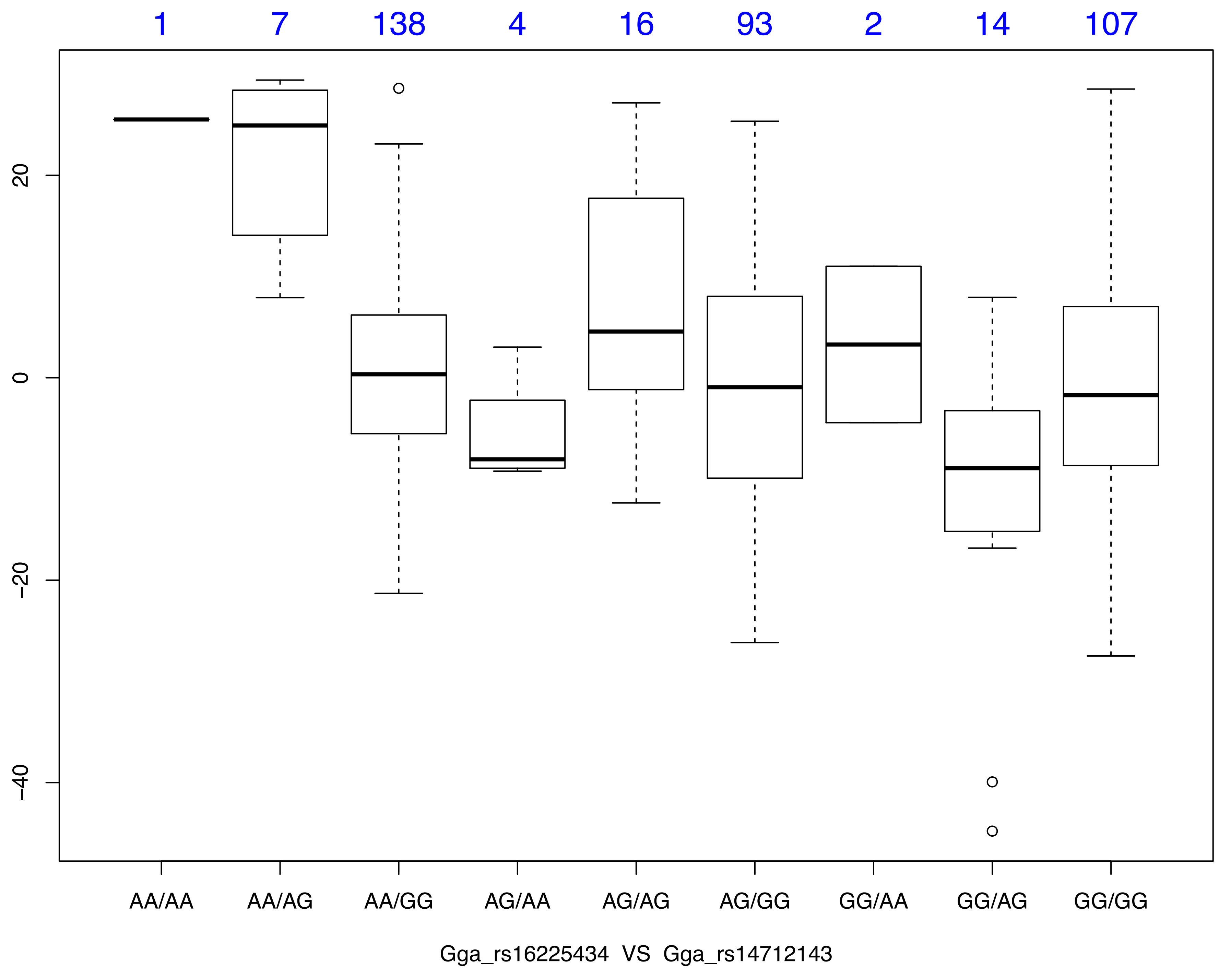
1
7
138
4
16
93
2
14
107
20
0
−20
−40
AA/AA
AA/AG
AA/GG
AG/AA
AG/AG
AG/GG
GG/AA
GG/AG
GG/GG
Gga_rs16225434 VS Gga_rs14712143

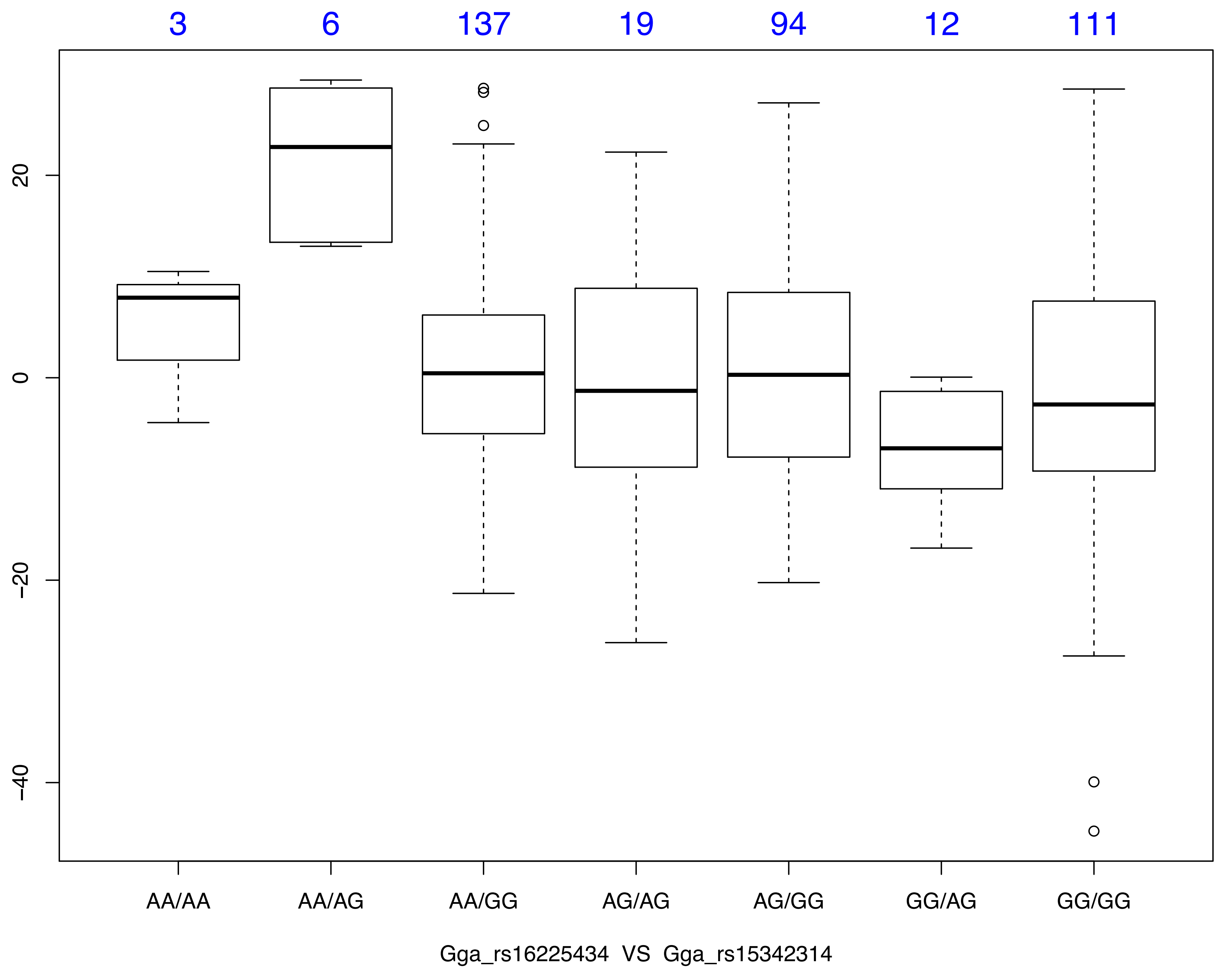

3
6
137
19
94
12
111
20
0
−20
−40
AA/AA
AA/AG
AA/GG
AG/AG
AG/GG
GG/AG
GG/GG
Gga_rs16225434 VS Gga_rs15342314

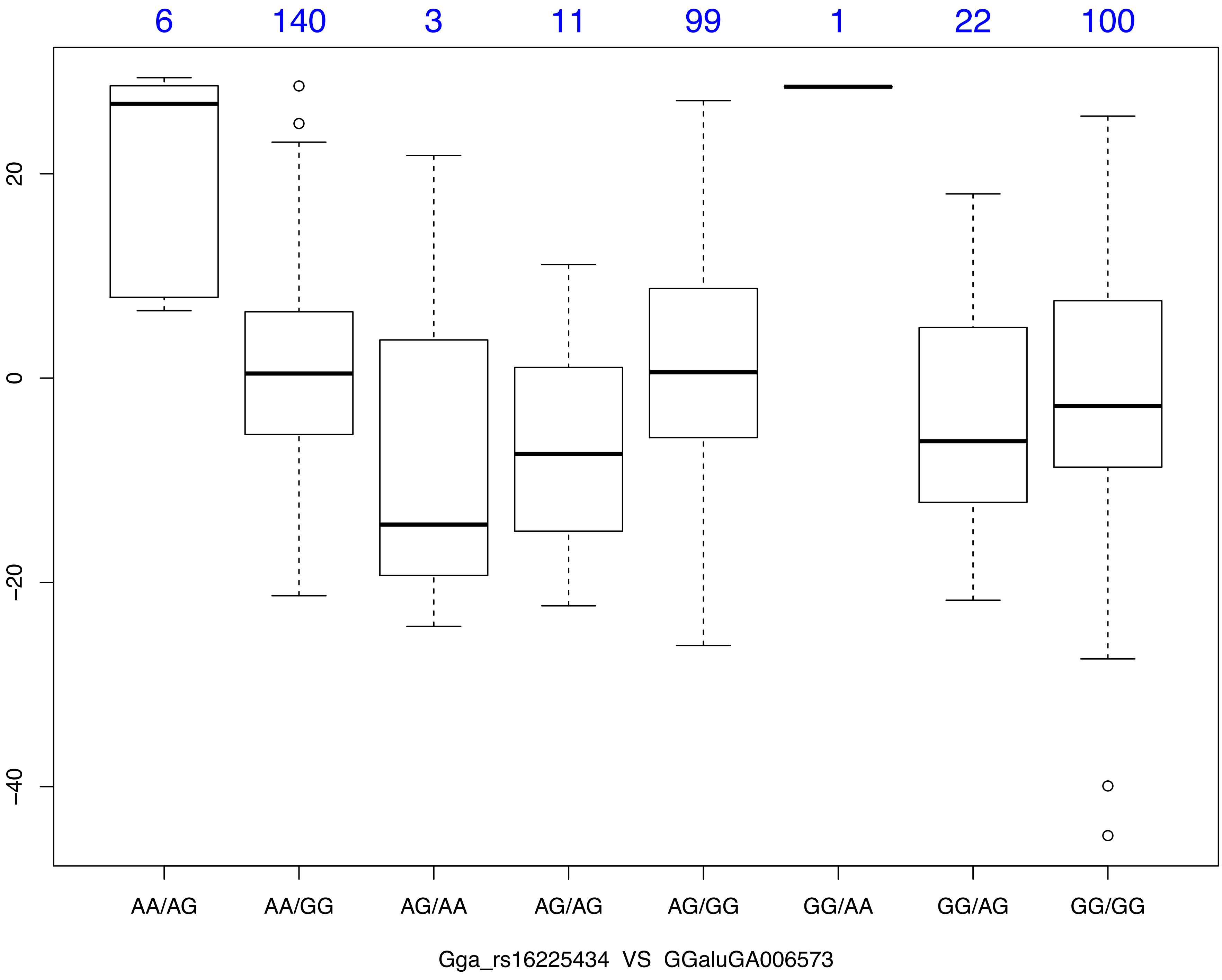
6
140
3
11
99
1
22
100
20
0
−20
−40
AA/AG
AA/GG
AG/AA
AG/AG
AG/GG
GG/AA
GG/AG
GG/GG
Gga_rs16225434 VS GGaluGA006573

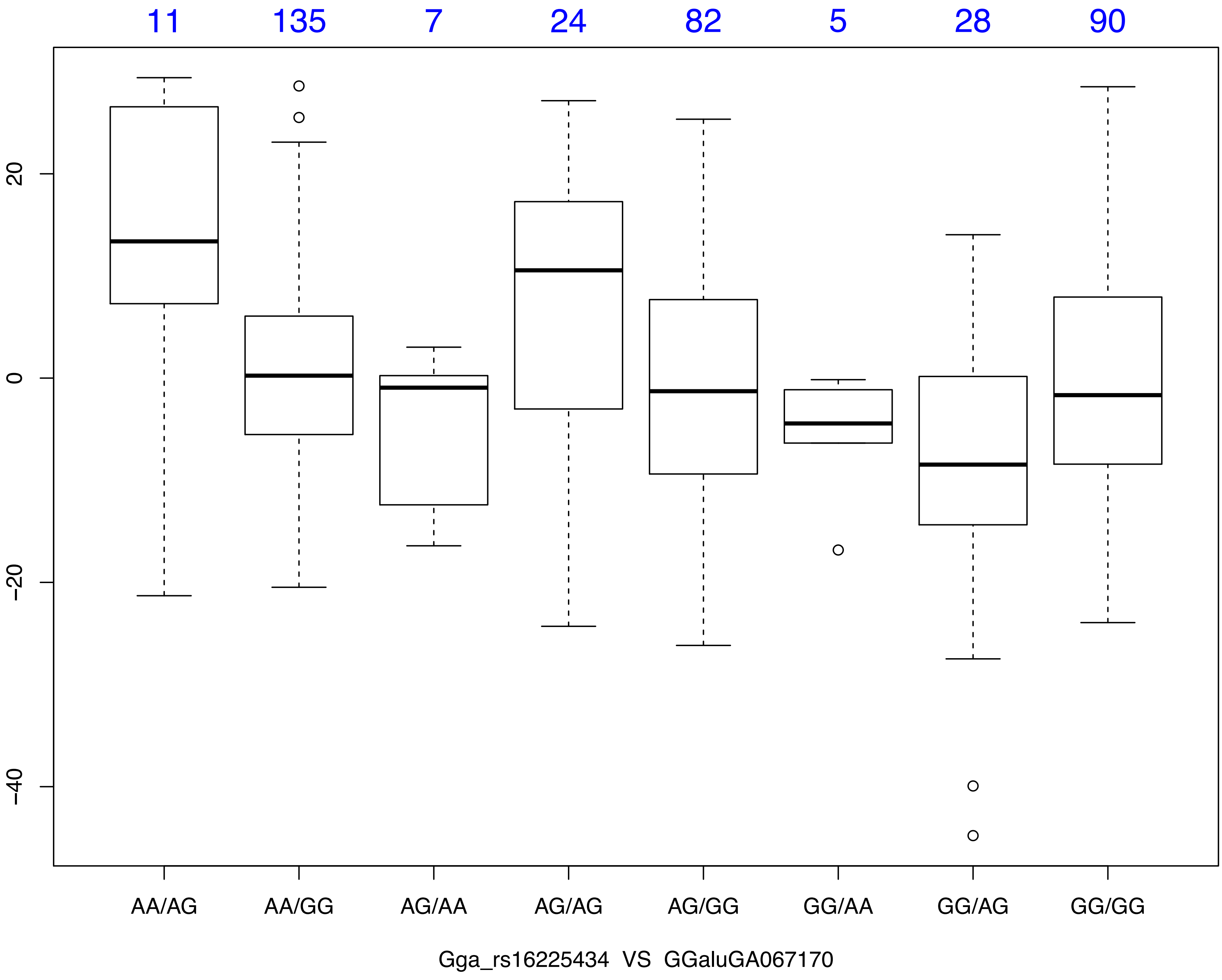

11
135
7
24
82
5
28
90
20
0
−20
−40
AA/AG
AA/GG
AG/AA
AG/AG
AG/GG
GG/AA
GG/AG
GG/GG
Gga_rs16225434 VS GGaluGA067170

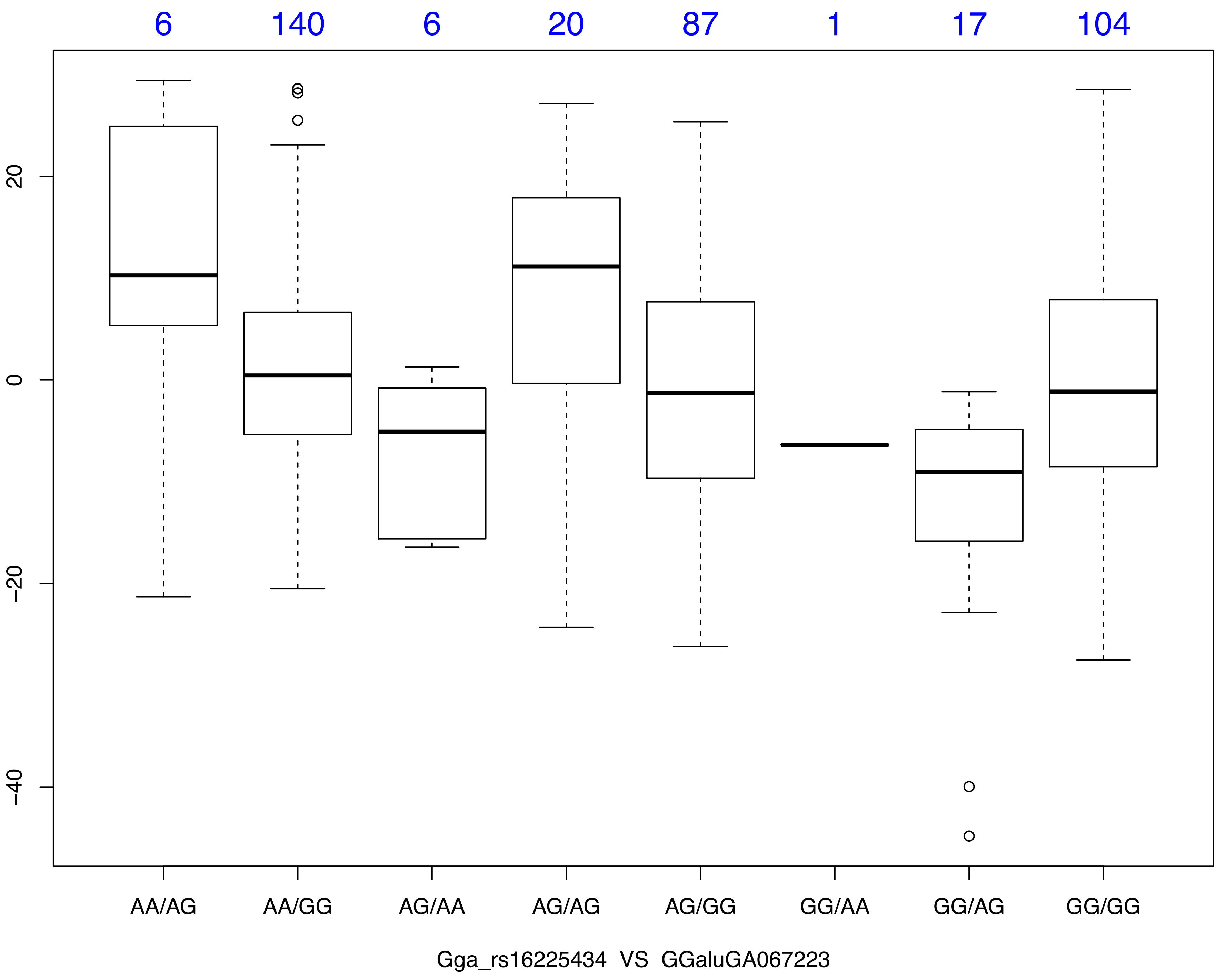

6
140
6
20
87
1
17
104
20
0
−20
−40
AA/AG
AA/GG
AG/AA
AG/AG
AG/GG
GG/AA
GG/AG
GG/GG
Gga_rs16225434 VS GGaluGA067223

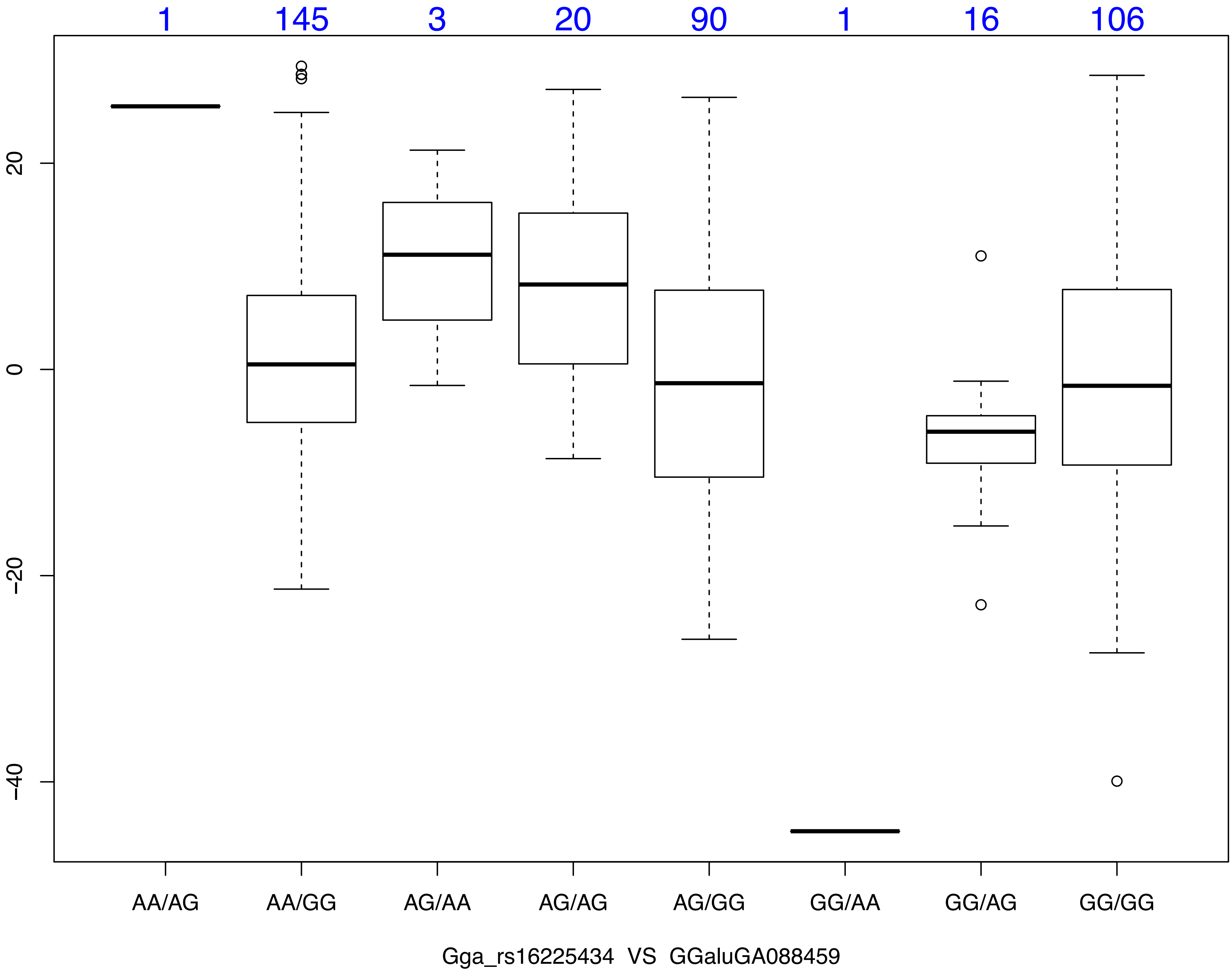

1
145
3
20
90
1
16
106
20
0
−20
−40
AA/AG
AA/GG
AG/AA
AG/AG
AG/GG
GG/AA
GG/AG
GG/GG
Gga_rs16225434 VS GGaluGA088459

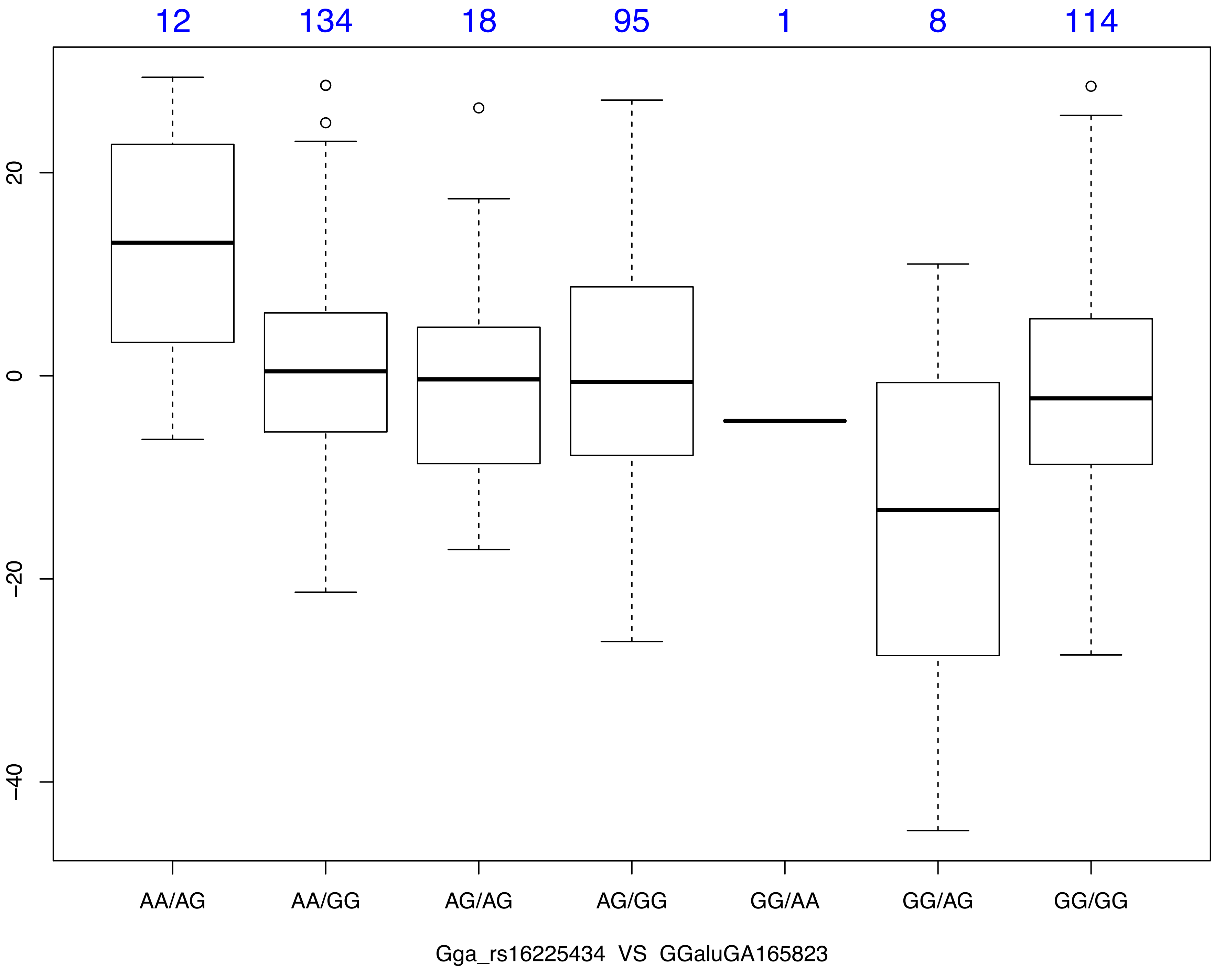

12
134
18
95
1
8
114
20
0
−20
−40
AA/AG
AA/GG
AG/AG
AG/GG
GG/AA
GG/AG
GG/GG
Gga_rs16225434 VS GGaluGA165823

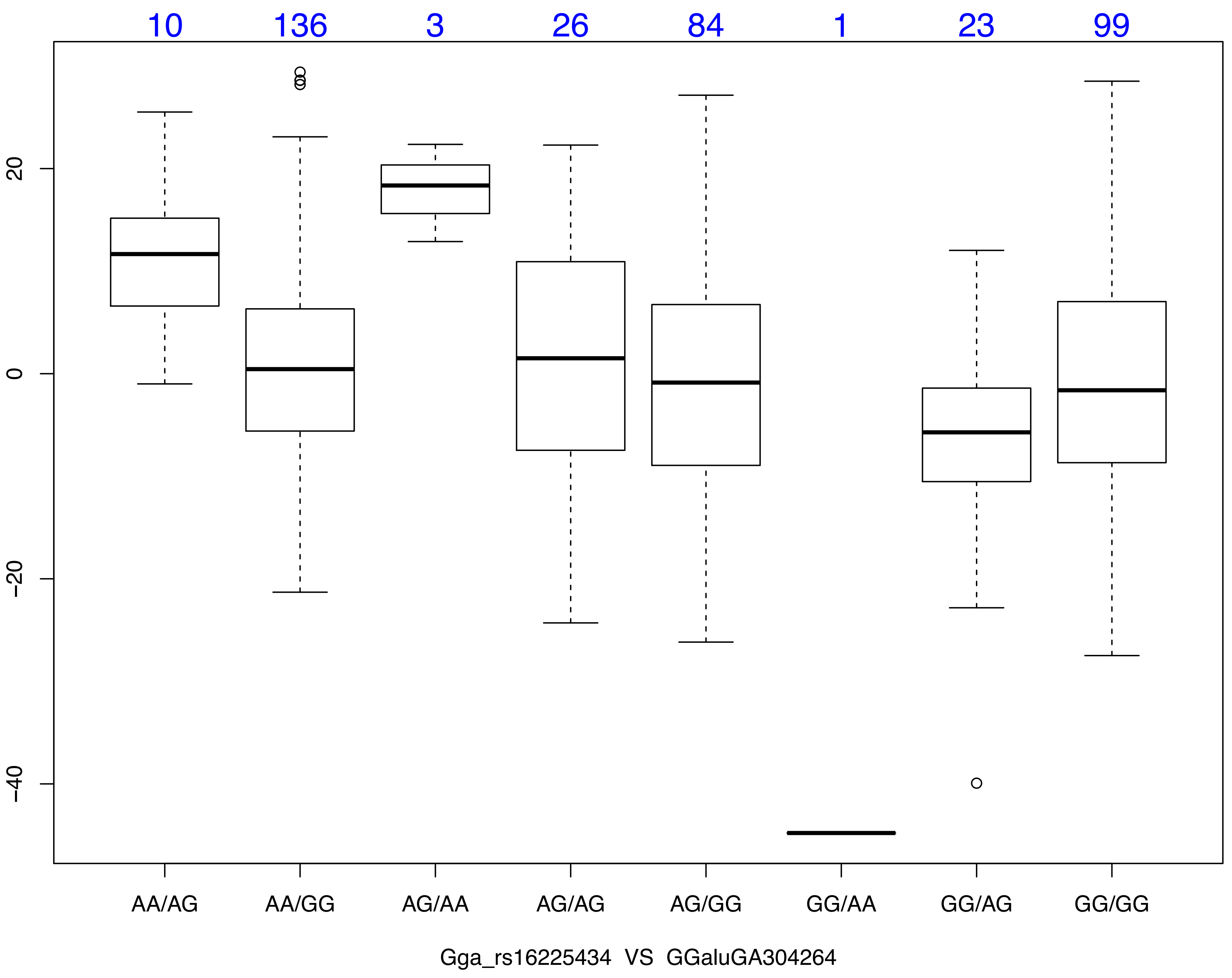

10
136
3
26
84
1
23
99
20
0
−20
−40
AA/AG
AA/GG
AG/AA
AG/AG
AG/GG
GG/AA
GG/AG
GG/GG
Gga_rs16225434 VS GGaluGA304264

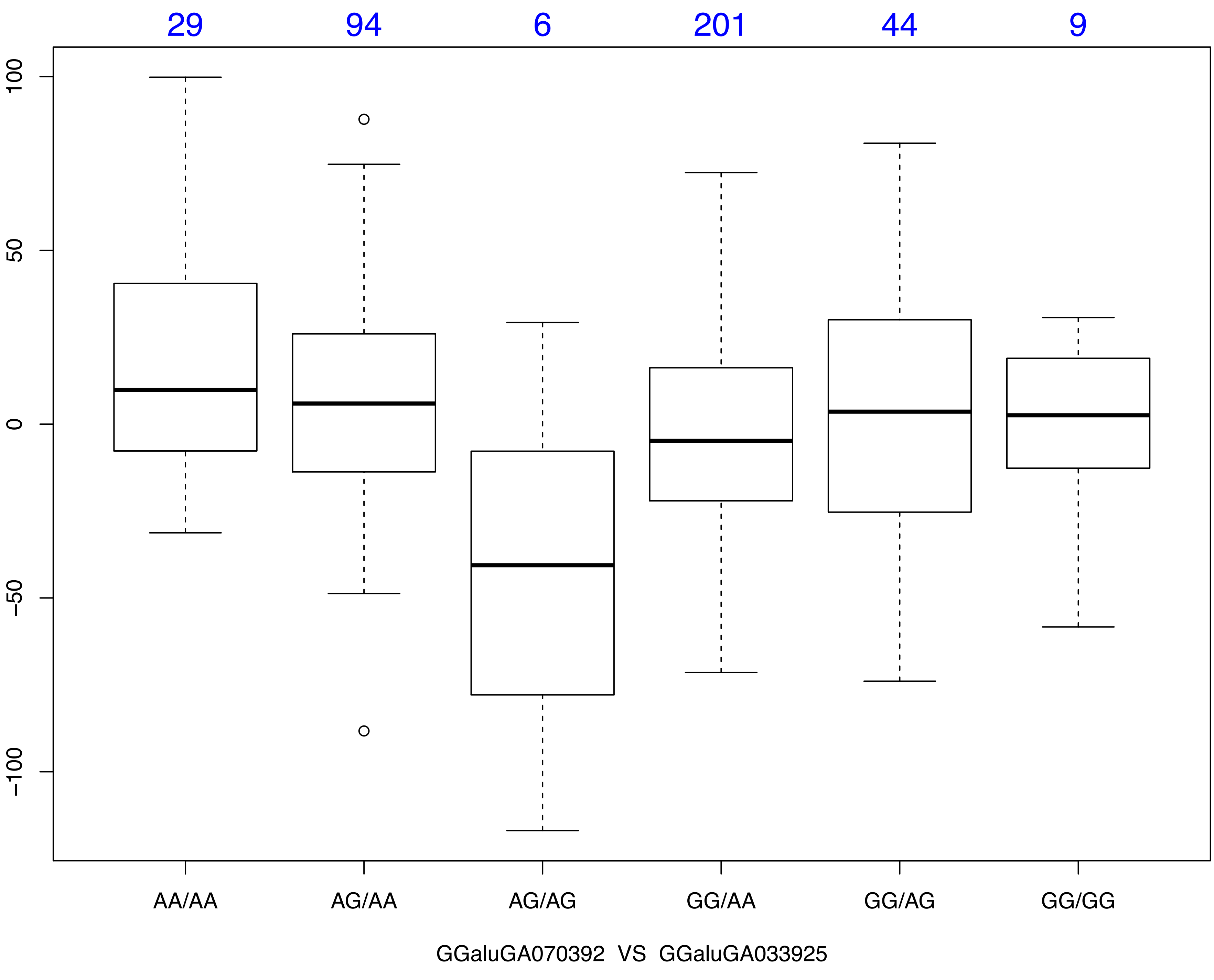
29
94
6
201
44
9
100
50
0
−50
−100
AA/AA
AG/AA
AG/AG
GG/AA
GG/AG
GG/GG
GGaluGA070392 VS GGaluGA033925

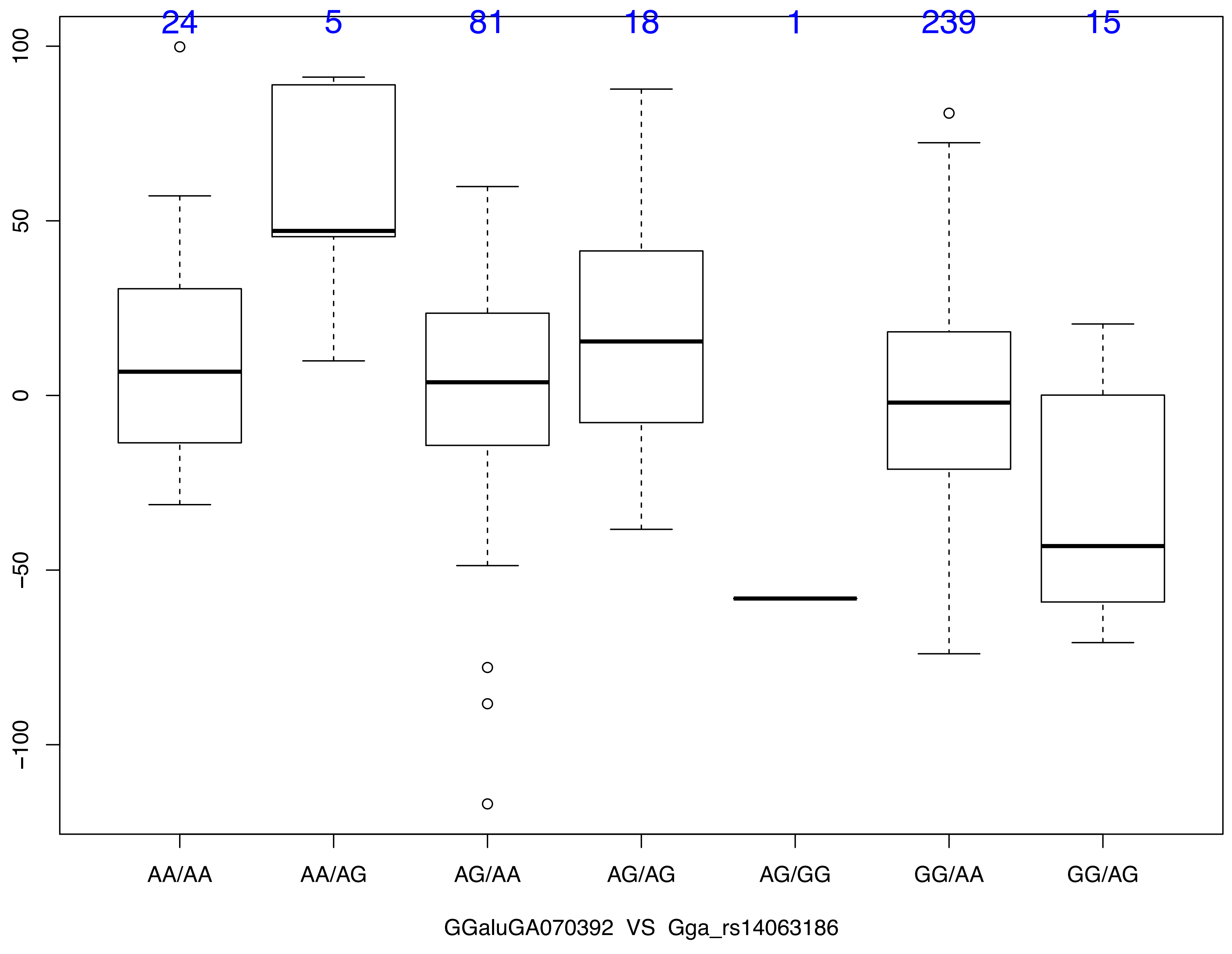
24
5
81
18
1
239
15
100
50
0
−50
−100
AA/AA
AA/AG
AG/AA
AG/AG
AG/GG
GG/AA
GG/AG
GGaluGA070392 VS Gga_rs14063186
