# Supplementary material for: Polygenic Basis and Variable Genetic Architectures Contribute to the Complex Nature of Body Weight —A Genome-Wide Study in Four Chinese Indigenous Chicken Breeds
Source: Front Genet. 2018 Jul 2;9:229. doi: 10.3389/fgene.2018.00229 (PMC6036123; doi:10.3389/fgene.2018.00229)
Supplement: DATA SHEET S1 — The R code for transforming the raw phenotypes for the vGWAS analysis. [file Data_Sheet_1.PDF]

The following R code can be used to transform the raw phenotypes for the vGWAS analysis:

```
zscore.yang <- function(raw_phenotype) qnorm((rank(raw_phenotype, na.last = "keep") -
0.5)/sum(!is.na(raw_phenotype)))
```
